# Phase Equilibria and Morphology of the TPU–Tactix–DGEBA Ternary System: Interference Approach and SEM Analysis

**DOI:** 10.3390/polym18202605

**Published:** 2026-10-21

**Authors:** Artem D. Ponomarenko, Irina O. Plyusnina, Uliana V. Nikulova, Aleksey V. Shapagin

**Affiliations:** Frumkin Institute of Physical Chemistry and Electrochemistry, Russian Academy of Sciences (IPCE RAS), 31-4, Leninsky prospect, 119071 Moscow, Russia; ponomarenhik@gmail.com (A.D.P.); irinaplyusninar@yandex.ru (I.O.P.); ulianan@rambler.ru (U.V.N.)

**Keywords:** tri- and difunctional epoxy resins, thermoplastic polyurethane, phase morphology, polymer blend, phase separation, ternary phase diagram, optical interferometry, interdiffusion

## Abstract

Combining epoxy oligomers with various functionality and thermoplastic modifiers enables targeted regulation of the architecture of polymer networks and the phase structure of composites. A comprehensive interference–microscopic approach is proposed for studying phase equilibria, diffusion characteristics, and structure formation kinetics in the ternary system thermoplastic polyurethane (TPU)–trifunctional epoxy oligomer (Tactix)–difunctional epoxy oligomer (DGEBA) cured with 4,4′-diaminodiphenylsulfone (DDS). It may provide control of the morphology of epoxy compounds in a narrow concentration range without changing the chemical nature of the components. The TPU–Tactix–DGEBA system is found to be thermodynamically compatible over a wide temperature range. A ternary diagram is constructed, serving as a concentration map of the final morphologies. Four topological regions were experimentally identified and described. It has been shown that the addition of Tactix (more than 30 vol.%) accelerates the onset of thermodynamic incompatibility and shifts the point of phase separation to lower conversion levels (from 22% to 15%). It was established that the average domain size of the dispersed phase (from 0.15 to 0.6 µm) is controlled by the relationship between the time to reach thermodynamic incompatibility and the kinetic inhibition of diffusion as conversion increases. The proposed approach demonstrates the possibility of precisely altering the morphology of epoxy compounds within a narrow concentration range (within 10 vol.%) without altering the chemical nature of the initial components.

## 1. Introduction

The field of materials science for polymer composites is currently transitioning from highly specialized compositions to universal systems [1,2] capable of delivering a wide range of performance properties with minor changes in chemical composition. Such systems can optimize production processes, reduce the number of components, and improve the reproducibility of properties at an industrial scale. This challenge is particularly significant for epoxy binders, which are widely used in construction, electronics, and the aerospace industry, where a combination of high strength and crack resistance is essential.

Traditional approaches to modifying epoxy compounds rely on the introduction of various additives [3,4], which change the molecular structure and properties, which is associated with an increase in the formulation complexity. In this regard, a promising approach is one based on controlling the material’s phase structure without altering the chemical nature of the components, by varying their concentrations within a narrow range. Combining epoxy oligomers with different functionalities is an effective way to control network structure. For example, bifunctional epoxy oligomers form networks with moderate density, while trifunctional ones provide higher glass transition temperatures [5,6]. Their combined use allows for control of network architecture and curing kinetics without the need for additional reactive additives and chemical reaction catalysts.

In most cases, the initial epoxy resin–thermoplastic system is homogeneous, but the curing reaction can result in the formation of a heterogeneous structure of a specific type, determined by the kinetics of the chemical curing reaction, the interdiffusion of components, and the temperature–time conditions of curing. Thus, modifying epoxy resins with thermoplastics allows for variation in the phase structure and, consequently, the mechanical properties of epoxy systems, such as crack resistance [7].

The modification of epoxy resins with thermoplastics has been extensively studied in the literature [8,9,10]. However, most studies focus on specific compositions without considering the possibility of deliberately forming different types of phase structures. For instance, study [11] examined a three-component system consisting of a reactive polymer and two thermoplastics without attempting to construct a phase diagram. In contrast, the authors of studies [12,13] obtained ternary phase diagrams for epoxy resin–thermoplastic–thermoplastic systems. These works are united by the desire to study phase separation by systematically varying compositions, with a focus on the study of three-component systems. The authors of study [14] obtained a three-component phase diagram using various techniques, including optical interferometry. However, when studying three-component systems, researchers lack a comprehensive approach that links phase equilibria in ternary curing systems with the final phase structure. It should also be noted that most authors consider binary epoxy oligomer–thermoplastic systems, examining the influence of the functionality of an individual epoxy component on phase separation processes and material properties. Meanwhile, the use of combinations of epoxy components with different functionalities can allow for varying the network density, which affects not only heat resistance but also the phase structure of the material.

Rigid-chain thermoplastics are the most common thermoplastic modifiers for epoxy blends. However, representatives of another class of polymers, such as thermoplastic polyurethane, are often overlooked. Incorporating thermoplastic polyurethane into epoxy blends enhances strength and crack resistance [15,16,17] and can also impart self-healing properties to the cured epoxy resin–TPU system [18].

Thus, an analysis of the presented literature data shows that the primary focus in studying the formed phase structure in epoxy–thermoplastic systems is binary thermoset–thermoplastic systems. For three-component systems, determining the homogeneous and heterogeneous regions is typically accomplished through experimental selection of compositions followed by examination of the resulting structure. Therefore, a methodological approach to determining the boundaries of the homogeneous and heterogeneous regions in three-component thermoset–thermoset–thermoplastic systems, with the goal of narrowing the concentration ranges for predicting the phase organization of the systems, remains insufficiently developed.

This work proposes an approach based on optical interferometry to determine the boundaries of homogeneous and heterogeneous regions within the concentration field of the Gibbs triangle of the phase diagram of the ternary system “bifunctional epoxy oligomer–trifunctional epoxy oligomer–thermoplastic modifier” during curing. The heterogeneous region thus defined is scanned by an electron microscope in concentrations to construct a ternary structural diagram showing the concentration ranges of various types of formed phase structures. The proposed approach aims to provide a technological tool for controlling the final phase structure in epoxy systems at a given temperature over a narrow concentration range without altering the chemical nature of the components.

## 2. Materials and Methods

### 2.1. Materials

**Figure 1:**
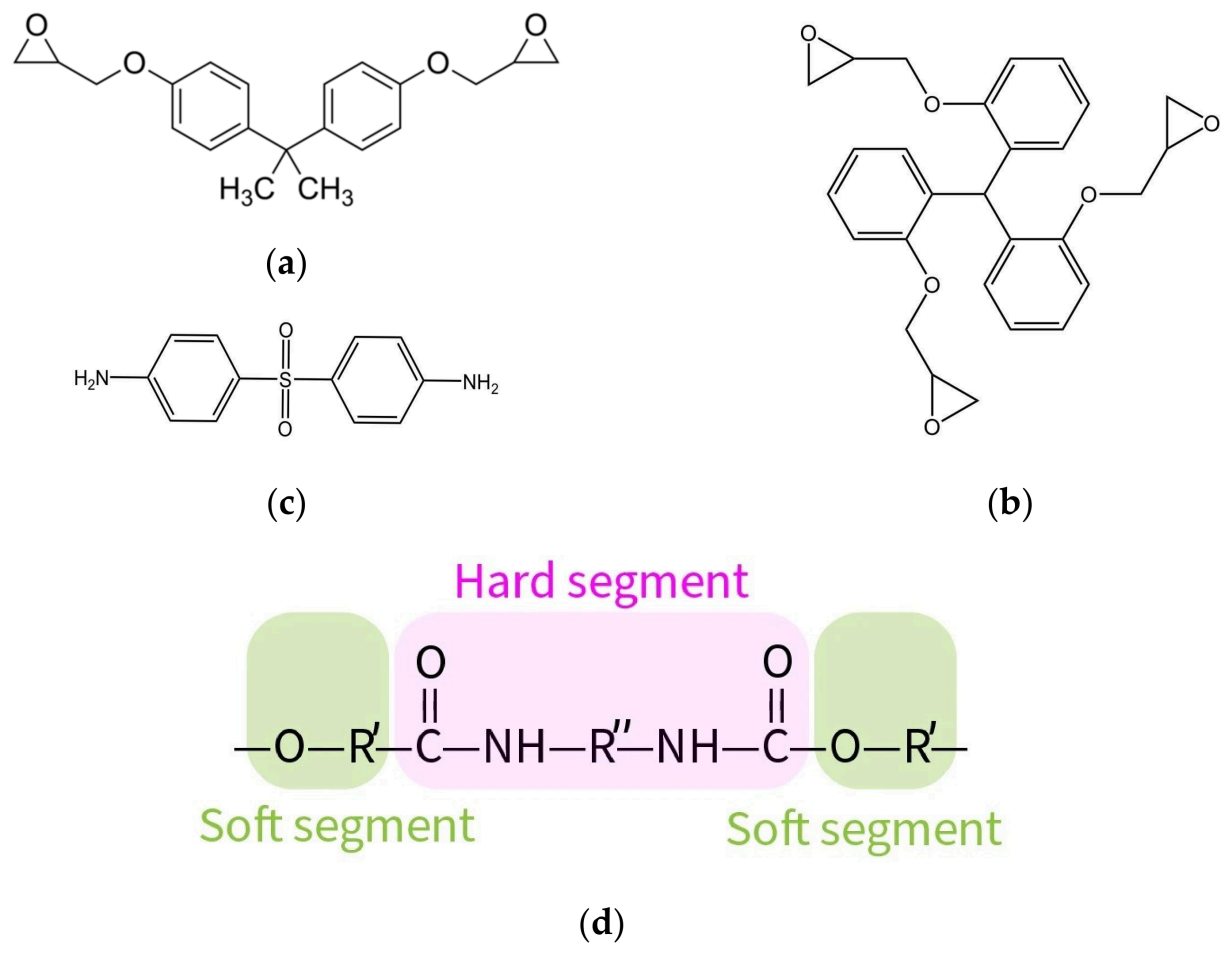
Structural chemical formulas: (**a**) DGEBA, (**b**) Tactix, (**c**) DDS and (**d**) generalized TPU structure [19], where R′ is the polyol segment and R″ is the chain extender.

Diglycidyl ether of bisphenol A (DGEBA) of the KER 828 brand was used (KUMHO P&B Chemicals, Gwangju, Republic of Korea), with Epoxide Equivalent Weight (EEW) 185–190 g/eq. Triglycidyl ether of triphenolmethane (Tactix) of the Tactix-742 brand was used (Huntsman Corporation, Woodlands, TX, USA), with EEW 150–170 g/eq. Thermoplastic polyurethane (TPU) of the MK 1185 brand was used, which is a commercial product (MK-Polymer, Seversk, Russia). The following was used as a hardener: aromatic amine 4,4′-diaminodiphenylsulfone (DDS) of the Aradur 9664-1 brand (Huntsman Corporation, Woodlands, TX, USA). The structural chemical formulas of DGEBA, Tactix, and DDS are shown in Figure 1. Due to the commercial nature of TPU, its exact chemical formula is not disclosed; therefore, a generalized structure of thermoplastic polyurethane is presented.

**Figure 2:**
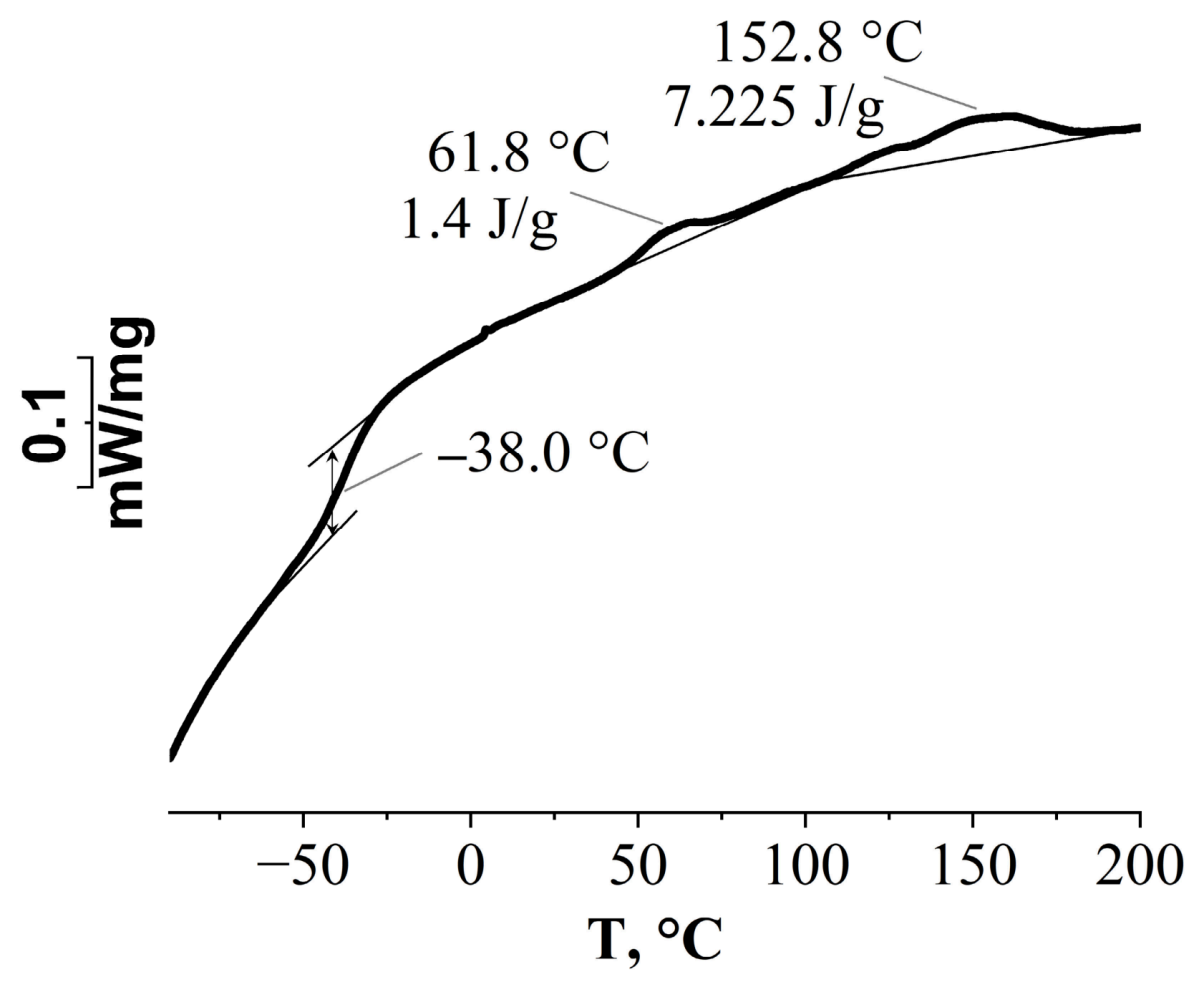
DSC thermogram for TPU brand MK1185, scanning speed 10 K/min, and primary heating.

TPU is characterized by a glass transition temperature (T_g_) of about −38 °C. In addition, TPU exhibits an endothermic peak associated with the melting of hard segments, in the temperature range from 90 to 160 °C (Figure 2). These data correlate well with [20].

### 2.2. Methods

#### 2.2.1. Optical Interferometry (OI)

Mass transfer processes in binary and quasi-binary gradient solutions were studied using optical interferometry on an ODA-2 (IPCE RAS, Moscow, Russia). Optical interferometry is based on the analysis of the interference picture formed when a refractive index gradient arises during the interdiffusion of components in a wedge-shaped gap (angle ≤ 2°) between two glasses, when passing a laser beam with λ = 635 nm [21]. To achieve this interference effect, the inner surfaces of the glasses are coated with a translucent layer of a highly reflective Ni-Cr alloy. Recording interferograms over time at various temperatures (temperature maintenance accuracy ±0.5 °C) allows for the construction of concentration profiles, determination of the concentration dependences of interdiffusion coefficients, and calculation of the diffusion activation energy.

In the diffusion region of interferograms recorded during the mixing of mutually soluble components, an S-shaped curvature of the interference lines is observed; the mathematical description of these lines corresponds to the concentration profile. The position of the Matano–Boltzmann plane on the concentration profile was determined as the concentration point where the diffusion fluxes of the components into each other are equal. Mutual diffusion coefficients were calculated from the obtained concentration distribution profiles using the Matano–Boltzmann method in accordance with Equation (1) [22].

(1) $$D_{v}(\varphi )=\frac{1}{2t}\frac{dx}{d\varphi }\int_{0}^{\varphi }\left(x_{M}-x\right)d\varphi$$

The resolution of the method was determined by the number of interference lines in the diffusion zone, which can also be calculated using Equation (2), where $n_{1}$ and $n_{2}$ are the refractive indices of the components, N is the total number of interference lines, and Δ is the increment of refractive index and concentration per line:

(2) $$N=\frac{n_{1}-n_{2}}{\Delta }$$

For an angle of θ = 2°, the refractive index increment is 0.003. The uncertainty in determining the position of the interference lines was ±1–2 pixels.

This method also assesses the thermodynamic compatibility of components and identifies regions of homogeneity/heterogeneity over a wide temperature and concentration range. A detailed diagram of the optical interferometer is presented in [4,23,24].

#### 2.2.2. Fourier Transform Infrared Spectroscopy (FTIR)

FTIR spectra of the initial Tactix, TPU, and their blends with varying TPU contents (without curing agent) were obtained on a Nicolet iN10 FTIR microscope (Thermo Scientific, Waltham, MA, USA) in ATR mode using a germanium crystal. Three FTIR spectra were recorded for each sample. Each spectrum was generated by accumulating 128 scans with a resolution of 4 cm^−1^ in the spectral range of 4000–675 cm^−1^ at 20 °C. Each spectrum was processed with automatic baseline correction using Omnic 9 software (Thermo Scientific, Waltham, MA, USA).

The characteristic absorption band ranges for TPU and Tactix are presented in Table 1.

#### 2.2.3. Differential Scanning Calorimetry (DSC)

Thermal properties of TPU and curing processes of TPU–Tactix+DDS mixtures with different TPU contents were studied using differential scanning calorimetry on a Netzsch DSC 204F1 Phoenix (Netzsch-Gerätebau GmbH, Selb, Germany). Sample preparation was performed in accordance with the international standards ISO 11357-3 [25] and ISO 11357-4 [26].

#### 2.2.4. Scanning Electron Microscopy (SEM)

The morphology of DDS-cured TPU–Tactix–DGEBA samples at 180 °C was studied using a scanning electron microscope JSM-6060A (JEOL, Tokyo, Japan). Preparation involved vacuum thermal spraying of a conductive carbon layer onto the surface of a low-temperature cleavage of the test object using a tabletop sprayer DSR1 (NanoStructured Coatings Co., Tehran, Iran). SEM images were obtained using secondary electrons at an accelerating voltage of 15 kV.

#### 2.2.5. Sample Preparation

1. To study the thermodynamic compatibility of the components using optical interferometry, binary Tactix–DGEBA mixtures of a given composition were prepared. Mixing was carried out at 120 °C, followed by an interferometry experiment.

2. Studying the compatibility of thermoplastics with pre-cured epoxy oligomer adducts enables various stages of curing to be simulated and changes in thermoplastic–thermoset compatibility to be tracked as the reaction progresses. Adducts of the Tactix–DGEBA epoxy mixture with a specified calculated conversion degree α were pre-formed, after which their compatibility with TPU was studied using optical interferometry.

Tactix–DGEBA adducts were prepared by mixing the components at 120 °C, followed by the addition of DDS in varying amounts relative to the stoichiometric content of epoxy groups (Supplementary Materials, Tables S1 and S2). The choice of the DDS amount enables systems with the specified calculated α values to be obtained. In the first stage, a series of compositions with calculated α values from 10 to 30% in 5% increments were prepared. After complete dissolution of the hardener, the adduct series was cured at 180 °C for 240 min. Since the conversion degree of the adducts used is less than the conversion degree at the gel point, which indicates sufficiently good mobility of the components for all amino groups of the curing agent to enter into a chemical reaction, the calculated value of the DDS concentration (Supplementary Materials, Equation (S3)) was used to obtain an epoxy system adduct with a specified conversion degree.

The resulting pre-cured adducts were sequentially tested in contact with TPU using optical interferometry. A continuous concentration profile formed in the interdiffusion zone, indicating the continued compatibility of the components at the corresponding α value, while the appearance of a phase boundary indicated the onset of phase decomposition. When a phase boundary appeared at a specific α value (from 10 to 30% in 5% increments), the α value was further refined. For this purpose, additional compositions were prepared with successive changes in the calculated degree of conversion in 1% increments and their compatibility with TPU was re-tested using optical interferometry. The α_pd_ value was taken to correspond to the lowest α value studied, at which a phase boundary was first observed in the interdiffusion zone.

3. The curing temperature of 180 °C for the TPU–Tactix–DGEBA mixture and the choice of DDS hardener were matched to the temperature regime of TPU, which ensures high macromolecular mobility and an efficient curing reaction. Preparation of the cured TPU–Tactix–DGEBA ternary systems involved pre-homogenization at 160 °C, followed by the addition of DDS at 120 °C and its complete dissolution. The temperature then increased to 180 °C, and curing was carried out for 360 min. After curing at 180 °C, the samples were cooled to room temperature inside the oven with the heating turned off, without forced cooling or rapid temperature changes. The compositions of the systems under study and the corresponding SEM images are presented in Supplementary Materials, Table S4.

## 3. Results

Analysis of the initial state of the system, specifically assessing the thermodynamic compatibility of the components, determining the interdiffusion coefficients, and determining the diffusion activation energy, is an important step before studying the curing processes and structure formation in the TPU–Tactix–DGEBA+DDS ternary system. The phase diagram, as a quantitative representation of the thermodynamic compatibility of the components, indicates the temperature–concentration ranges for the formation of a homogeneous or heterogeneous structure before the onset of the chemical curing reaction, while the diffusion constants and their evolution during curing enable the correct interpretation of curing kinetics and structure formation processes.

### 3.1. Phase Equilibria and Interdiffusion in the TPU–DGEBA, TPU–Tactix, Tactix–DGEBA Systems

To assess the compatibility of the initial components via diffusion studies, interferograms were obtained in the temperature range of 80–200 °C with 20 °C steps. S-shaped interference lines were identified in the diffusion zone, indicating the optical transparency of the system and the absence of phase structures larger than ~300 nm (λ/2); this signifies complete mutual solubility of the contacting components. Upon a change in temperature, the labile system reached equilibrium within a maximum of 15 min, as determined by the stabilization of the number of interference lines. The system’s homogeneity was maintained throughout the heating–cooling cycle. This allows us to conclude that the data regarding component compatibility were obtained under equilibrium conditions.

To confirm the homogeneity of the systems, additional DSC studies were carried out on the initial components of TPU, Tactix, and their blend TPU (33.3 vol.%)–Tactix (66.7 vol.%) (Supplementary Materials, Figure S1). It was found that only one glass transition temperature was identified in the thermogram of the TPU–Tactix blend system. Furthermore, the glass transition temperature of the blended composition (6.4 °C) corresponds to the value (3.5 °C) calculated using the Fox additive equation (Equation (3)).

(3) $$\frac{1}{T_{g}}=\frac{\omega_{1}}{T_{g,1}}+\frac{\omega_{2}}{T_{g,2}}$$

Thus, the DSC studies of the mixed composition confirm the homogeneity of the mixtures, determined on the basis of studies using the optical interferometry method.

**Figure 3:**
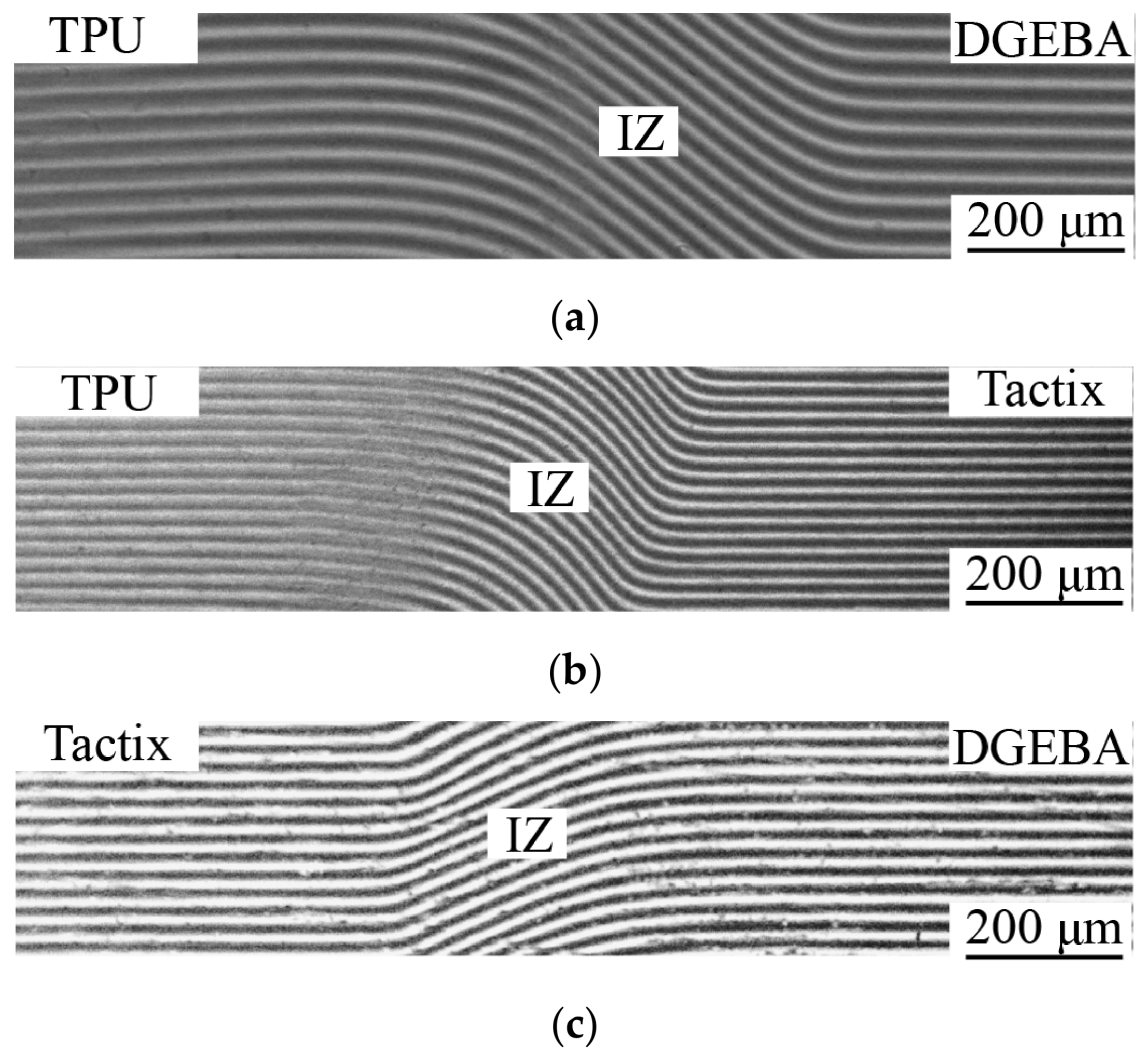
Interferograms of the interdiffusion zone of the systems: (**a**) TPU–DGEBA at 160 °C, 16 min; (**b**) TPU–Tactix at 180 °C, 25 min; (**c**) Tactix–DGEBA at 80 °C, 25 min. IZ is the interdiffusion zone.

When components of the TPU–DGEBA, TPU–Tactix, and Tactix–DGEBA systems come into contact (Figure 3), a spontaneously formed transition (interdiffusion) zone is observed in the area of their direct contact. Within this region, the composition and properties continuously change as they move from one component to another, due to interdiffusion processes. The composition distribution profiles within the interdiffusion zone (IZ) for all three binary systems are S-shaped curves transitioning from the parallel interference lines of one component to the interference lines of the other component.

**Figure 4:**
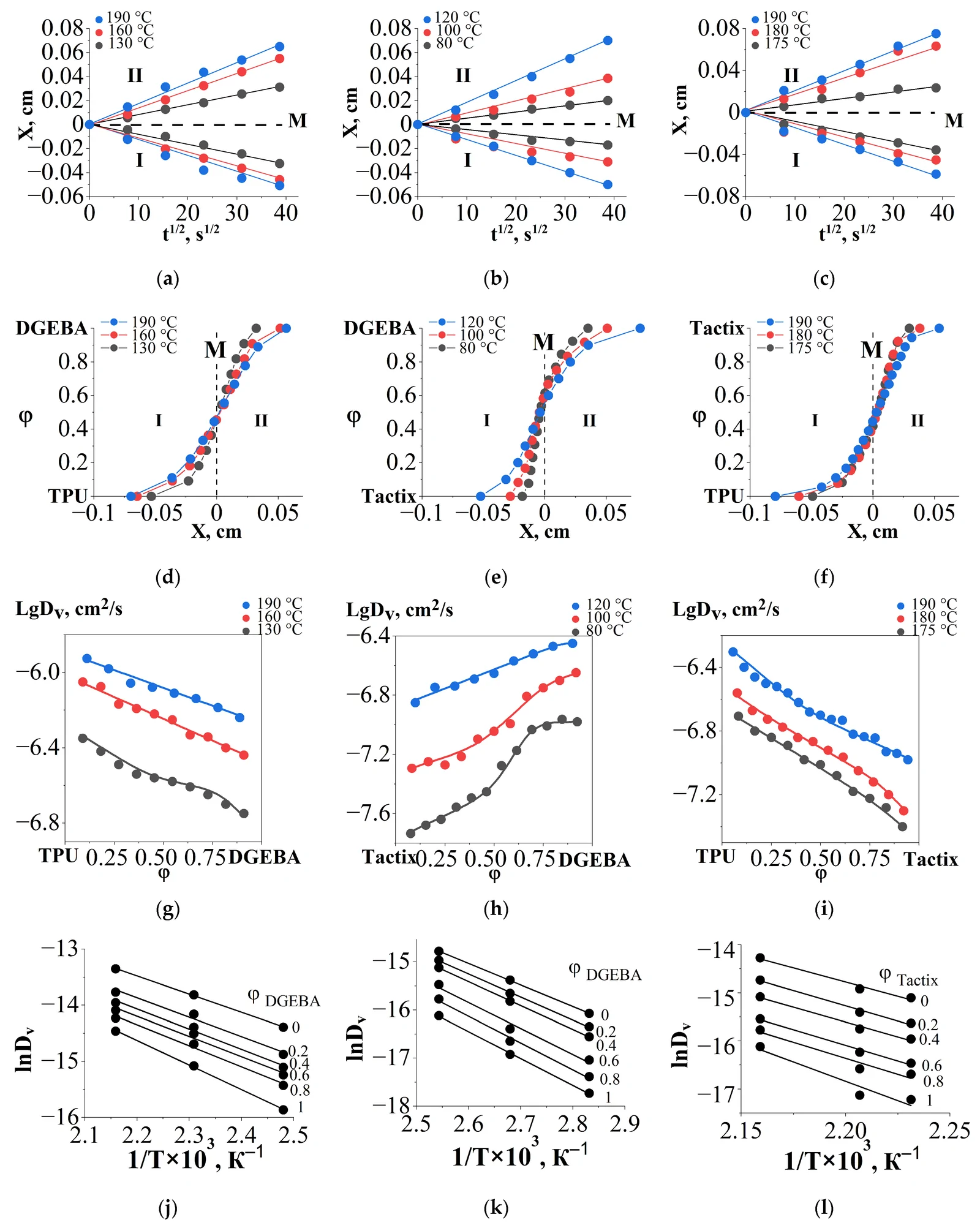
(**a**–**c**) Kinetic dependences of the motion of isoconcentration planes and concentration profiles of binary systems obtained at: (**d**) 16 min and (**e**,**f**) 25 min. (**g**–**i**) Concentration dependences of the interdiffusion coefficients; (**j**–**l**) temperature dependences of the diffusion coefficients for the systems (**a**,**d**,**g**,**j**) TPU–DGEBA; (**b**,**e**,**h**,**k**) Tactix–DGEBA; and (**c**,**f**,**i**,**l**) TPU–Tactix. M is the Matano–Boltzmann plane. Temperature values are shown in the figures. Diffusion flows: I – DGEBA in TPU (**a**,**d**); DGEBA in Tactix (**b**,**e**); Tactix in TPU (**c**,**f**); II – TPU in DGEBA (**a**,**d**); Tactix in DGEBA (**b**,**e**); TPU in Tactix (**c**,**f**).

Over time (t), the size of the diffusion zone (∆X) increases. According to diffusion theory, this relationship in coordinates ∆X = k√t, where k is a constant characterizing the partial diffusion coefficients of the components and X represents the coordinates of the plane in the interdiffusion zone, should be described by a linear relationship (Figure 4a–c). This type of movement of isoconcentration planes was observed across the entire selected temperature range. This indicates that when the components of the TPU–DGEBA, Tactix–DGEBA, and TPU–Tactix systems are mixed, no chemical reaction occurs; instead, the blending process is spontaneous and follows a diffusion mechanism.

In the interdiffusion zone, a continuous concentration profile is observed from one component to another. The position of the calculated Matano–Boltzmann plane is constant and corresponds to the middle region of the compositions (Figure 4d–f). Complete mutual solubility of the components is observed throughout the studied temperature range.

The continuous concentration profiles of the binary systems were used to calculate the interdiffusion coefficients via the Matano–Boltzmann method and to plot their concentration dependence (Figure 4g–i). To calculate the apparent activation energy of diffusion for each system, the concentration dependences of the interdiffusion coefficients were plotted for at least three temperatures. It is evident that with increasing experimental temperature, a consistent increase in the interdiffusion coefficients is observed (Figure 4j–l). Note that for the TPU–DGEBA system, a more significant decrease in the interdiffusion coefficients is observed as the temperature decreases to 130 °C. This is due to the restructuring of the supramolecular structure caused by the crystallization of rigid TPU segments and the deterioration of the mobility of macromolecules, which limits the diffusion mobility in the system.

**Figure 5:**
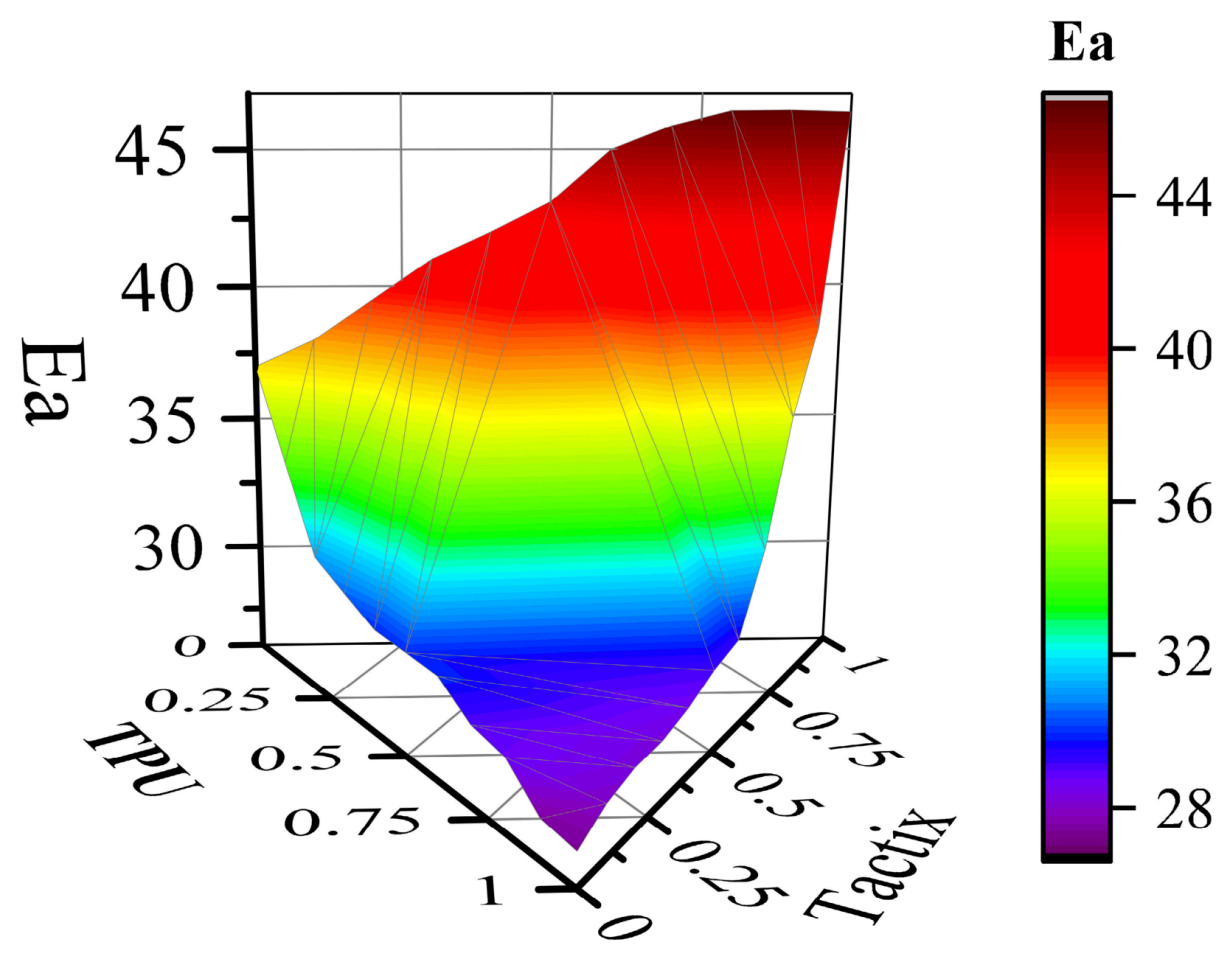
Three-dimensional concentration dependence of the apparent activation energy of interdiffusion in the coordinates of the composition of TPU–DGEBA, Tactix–DGEBA, TPU–Tactix.

Based on the temperature dependences of the diffusion coefficients (Figure 4j–l) and using the Arrhenius equation [24], the activation energies of the diffusion process were calculated (Figure 5). The obtained values of the activation energy of interdiffusion smoothly change from the values of the activation energy of self-diffusion of TPU (26.8 kJ/mol) to the values of Tactix (46.5 kJ/mol) and DGEBA (36.6 kJ/mol).

**Figure 6:**
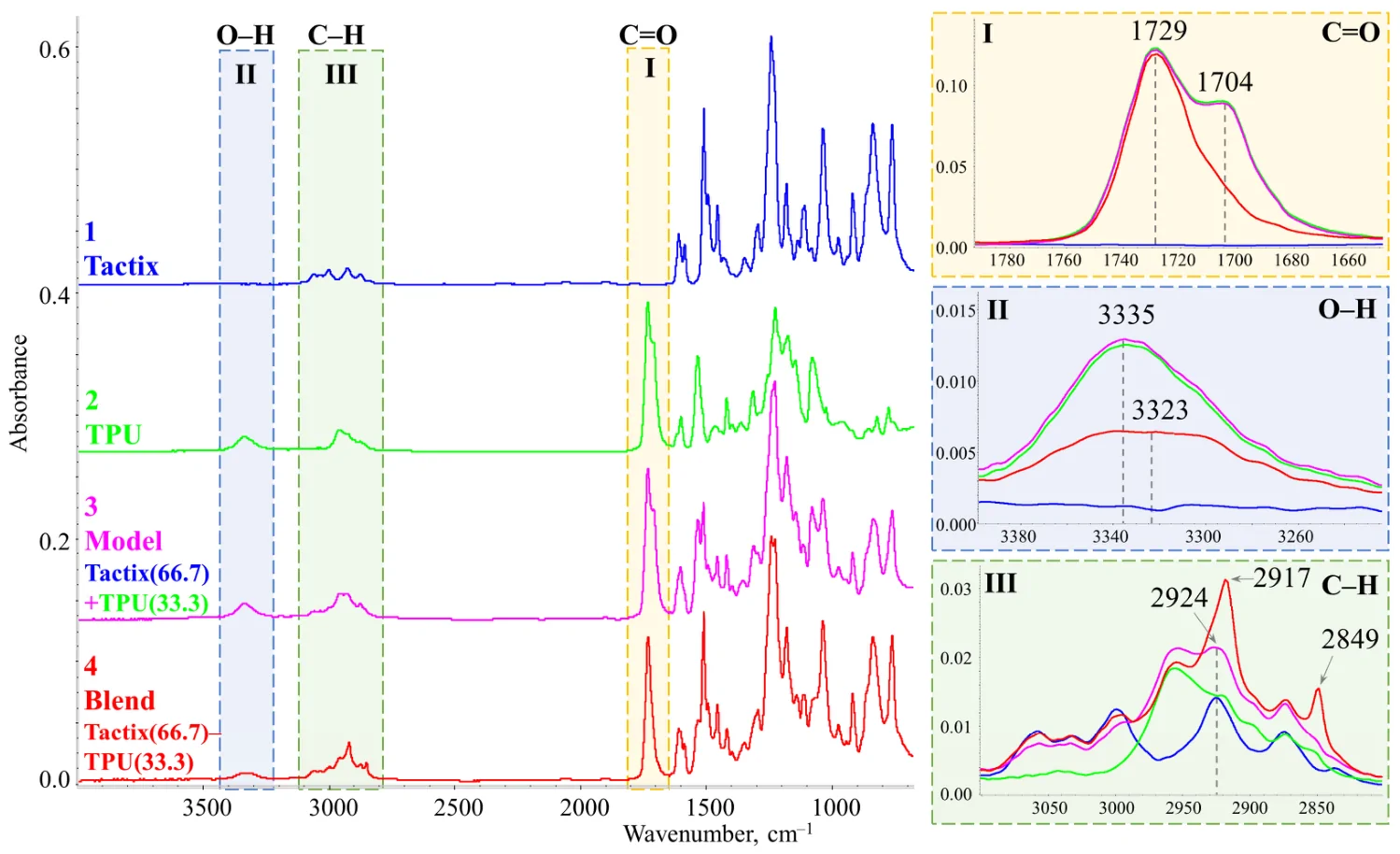
FTIR spectra of Tactix (spectrum 1) and TPU (spectrum 2); model spectrum of TPU (33.3 vol.%)–Tactix (66.7 vol.%) (spectrum 3); and real mixture of TPU (33.3 vol.%)–Tactix (66.7 vol.%) (spectrum 4). The insets with the overlap of these spectra display specific regions of the C=O (region I), O–H (region II), and C–H (region III) stretching bands.

**Figure 7:**
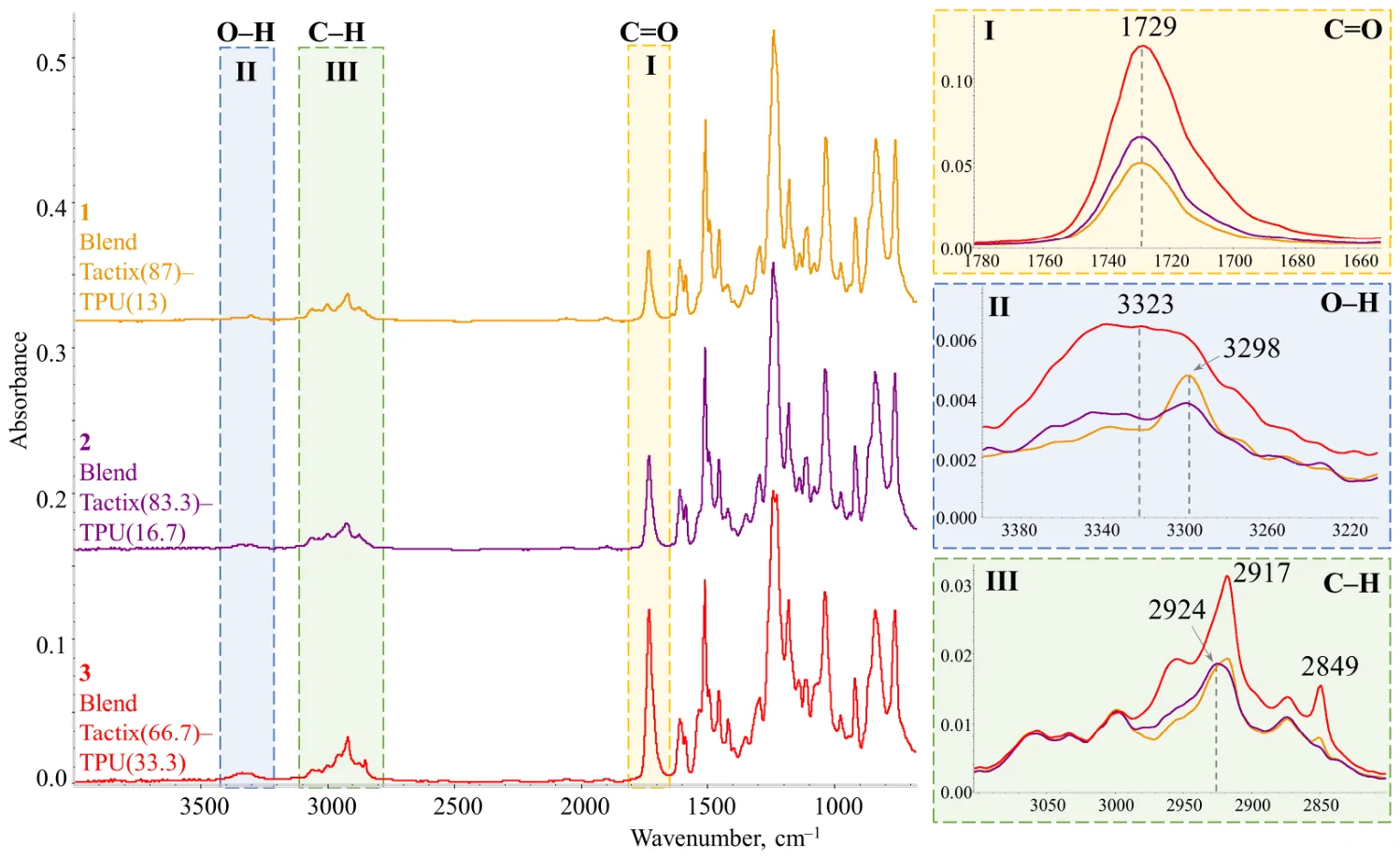
FTIR spectra of a mixture of TPU (13 vol.%)–Tactix (87 vol.%) (spectrum 1), a mixture of TPU (16.7 vol.%)–Tactix (83.3 vol.%) (spectrum 2), and a mixture of TPU (33.3 vol.%)–Tactix (66.7 vol.%) (spectrum 3). The insets with the overlap of these spectra display specific regions of the C=O (region I), O–H (region II), and C–H (region III) stretching bands.

To confirm the absence of a chemical reaction between Tactix and TPU, which is possible due to the presence of amide groups in the TPU structure, FTIR spectra of the initial components and their mixtures with a TPU content of 13, 16.7, and 33.3 vol.% were obtained (Figure 6 and Figure 7). The Tactix spectrum (blue) does not differ from the standard spectra for epoxy resin [27,28]. The TPU spectrum (green) is also typical [19,29]. The authors point out that the structure of thermoplastic polyurethanes is based on the alternation of hard and soft segments and chain extenders to link them. Hard blocks are polar rigid structures and are formed by the reaction of diisocyanates with low-molecular-weight chain extenders. 4,4′-Methylene diphenyl diisocyanate (MDI) is often used as a diisocyanate, and chain extenders are usually diols (e.g., 1,4-butanediol, ethylene glycol, etc.). Soft blocks of TPU are long, flexible oligomeric chains and are often polyethers or polyesters. According to the FTIR spectrum (Figure 6), TPU is an aromatic thermoplastic polyurethane with several important features. First, the characteristic peaks of the aromatic ring at 1597, 1530, and 815 cm^−1^ indicate a diisocyanate in the chemical structure. Secondly, the presence of absorption bands of stretching vibrations of C–O–C ether bonds in the region of 1200–1000 cm^−1^ indicates a polyether segment, and a broad absorption band of hydroxyl groups (superimposed on amide groups in the region of 3600–3100 cm^−1^) with stretching vibrations of aliphatic chains (2950–2860 cm^−1^) indicates the presence of a chain extender based on a short diol (e.g., 1,4-butanediol). Thirdly, most of the functional groups of TPU (which, in the studies presented above, most often concern rigid diisocyanate blocks) are involved in the formation of hydrogen bonds, since the C=O peak has a characteristic doublet (region I). The more intense left part of this (1729 cm^−1^) corresponds to free carbonyl groups (ether or urethane), while the right part (1704 cm^−1^) indicates carbonyls participating in hydrogen bonds of the type –N–H···O=C–.

Based on the spectra of the initial components, a model spectrum of TPU (33.3 vol.%)–Tactix (66.7 vol.%) (spectrum 3) (pink spectrum 3 in Figure 6) was obtained using the software’s built-in spectral addition function. This spectrum is used for comparison with the spectrum of a real TPU (33.3 vol.%)–Tactix (66.7 vol.%) blend (red spectrum 4 in Figure 6). The spectra of the initial components were preliminarily normalized with respect to the spectrum of the real mixture: the Tactix spectrum by the band at 1508 cm^−1^ (the internal standard for epoxy resin corresponding to the stretching vibrations of C=C bonds in the aromatic ring of bisphenol A [30]), and the TPU spectrum by the band at 1412 cm^−1^ (deformation vibrations of C–H bonds in the methylene groups –CH_2_– of diisocyanate [31]). The model spectrum was necessary to compare the position of its main characteristic absorption bands with the spectrum of the real mixture and to analyze potential structural changes in the system. It was found that the model spectrum, based on the position of the main characteristic bands, correlates well with the spectrum of the real mixture, which indicates the absence of a chemical interaction between Tactix and TPU. It should be particularly noted that no changes in the intensity or position of the absorption band at 914 cm^−1^ (corresponding to the epoxy ring) were observed for either of the studied mixtures. This indicates the absence of signs of epoxy ring opening and, consequently, the absence of a chemical reaction between the components. However, a number of important features were identified. On the one hand, the complete overlap of the absorption bands of the individual components indicates the homogeneity of the mixture. On the other hand, the differences between the model spectrum (3) and the spectrum of the mixture (4) indicate conformational changes in the TPU when mixed with the epoxy resin. Thus, region I (Figure 6) shows a superposition of the spectra for the absorption band of the C=O carbonyl group. It can be seen that the peak intensity at 1729 cm^−1^ (free carbonyl group) for the model and for the real mixture is almost the same and coincides with the same peak for pure TPU. However, the peak at 1704 cm^−1^ (hydrogen-bonded carbonyl group) is present in the model spectrum, as in the initial TPU, but is absent from the spectrum of the real blend. This indicates the disruption of hydrogen bonds in TPU upon blending with Tactix, resulting in the real blend being characterized by the presence of only free carbonyl groups. In region II (Figure 6), corresponding to the O–H hydroxyl groups and N–H groups, an absorption band is observed for both the initial TPU and the model spectrum, as well as for the real blend. However, in the latter case, it is less intense, and its center is slightly shifted to the right. For TPU, this peak primarily corresponds to N–H vibrations, and also partially corresponds to –N–H···O=C– hydrogen bonds. The decrease in band intensity for the real blend is due to a decrease in the proportion of hydrogen bonds within the TPU. However, the potential shift in the hypothetical midpoint of the peak to the right indicates that the actual TPU-Tactix blend either contains traces of sorbed moisture or contains a small number of hydrogen bonds, but not between TPU molecules. New hydrogen bonds may form between the TPU and Tactix molecules. Such interactions may occur between the isolated OH groups of the Tactix epoxy resin (typically present in small amounts) or between absorbed moisture and the polar NH groups of TPU. It should be noted that an additional experiment on moisture sorption by the mixture, carried out using the desiccator method over a humidity range of 0–100%, showed no significant change in either the overall spectrum or the peak in the 3400–3200 cm^−1^ region (experimental data are presented in Supplementary Materials, Figure S2). Region III (Figure 6) shows the range of 3000–2800 cm^−1^ (absorption bands of the –CH_2_– and –CH_3_ groups). It is evident that, compared to the initial components and the model, the spectrum of the real mixture has two sharp peaks at 2917 and 2849 cm^−1^, which are shifted to the right relative to the others. This also indicates that the TPU macromolecules in the epoxy resin medium unfold and transform from a dense block into individual chains surrounded by resin molecules. Meanwhile, the C–H vibration range of the aromatic ring (3100–3000 cm^−1^) remains virtually unchanged, indicating no changes in the conformational state of the benzene rings of the resin during mixing.

Figure 7 shows the effect of TPU content on its interaction with Tactix epoxy resin during mixing. All spectra were also normalized to the absorption band of 1508 cm^−1^. It is clear that the spectra of the mixtures for 13, 16.7, and 33.3 vol.% TPU correlate well with each other and differ primarily due to the concentration factor. In region I (Figure 7), the intensity of the 1729 cm^−1^ band increases consistently with increasing TPU content in the blend. It should be noted that for all compositions (13, 16.7, and 33.3 vol.% TPU), only one free carboxyl peak is observed. Region III (Figure 7) also contains the previously noted shifted peak at 2917 cm^−1^, characteristic of methyl groups; however, at low TPU content, its intensity decreases significantly. Meanwhile, the peak at 2849 cm^−1^ is virtually degenerate for blends with a low TPU content. This also confirms a smooth change in the conformational states of both the resin and TPU during blending with increasing TPU content. Region II (Figure 7) exhibits some distinctive features. Spectra 1 (yellow) and 2 (purple) for Tactix blends with TPU (13 vol.%) and TPU (16.7 vol.%) are close to the baseline, since at low TPU concentrations, the hydrogen bonds within the TPU are already broken. It should be noted that for the blend with TPU (13 vol.%), a small peak at 3298 cm^−1^ is more pronounced, which decreases for TPU (16.7 vol.%) and is completely transformed for TPU (33.3 vol.%). This indicates that at a low TPU content, one type of hydrogen bond begins to form (as mentioned earlier, it is possible that new hydrogen bonds may form between TPU and Tactix), which changes with an increase in the content of N–H groups in the blend; the absorption band becomes more intense, broader, and the center shifts to 3323 cm^−1^.

Thus, it was established that the components of the TPU–Tactix–DGEBA system are characterized by complete thermodynamic compatibility over the entire temperature range studied. Analysis of the concentration dependences of the interdiffusion coefficients and diffusion activation energies showed that with increasing temperature, the mobility of Tactix approaches the mobility of DGEBA, and at a temperature of 120 °C, the difference (ΔlgD_v_) is less than 0.5 [cm^2^/s].

### 3.2. Composition and Morphology of the Heterogeneous Region in the TPU–Tactix–DGEBA+DDS System

**Figure 8:**
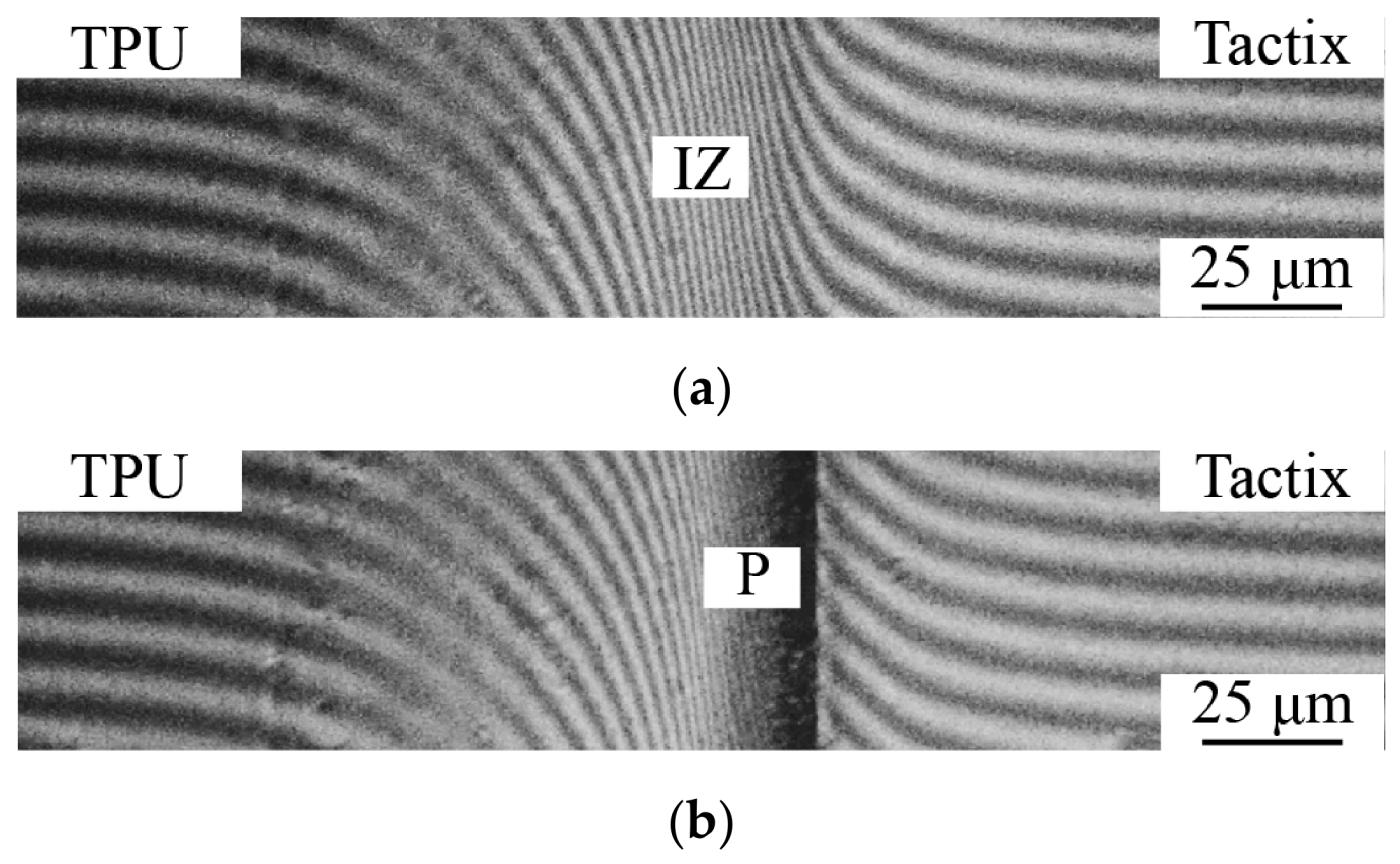
Interferograms of the interdiffusion zone during curing of the TPU–Tactix+DDS system at 180 °C: (**a**) 18 and (**b**) 24 min. IZ is the interdiffusion zone; P is the phase boundary.

In most thermoplastic–epoxy systems, the chemical curing reaction leads to the formation of a heterogeneous structure, which is caused by a deterioration in the thermodynamic stability of the system due to an increase in the molecular weight of the epoxy oligomer. Optical interferometry makes it possible to visually monitor this transition of a system from a homogeneous state to a heterogeneous one. Thus, in the interferogram of the TPU–Tactix+DDS system obtained after 18 min of reaction (Figure 8a), complete compatibility of the components is observed. As curing proceeds, a phase boundary (P) is formed in the interdiffusion zone (IZ), demonstrating the heterogeneity of the mixture in a certain concentration region (Figure 8b).

However, due to the kinetic properties of the curing reaction, differences in the density of the forming spatial network, and variations in the molecular weight of the initial components, it is possible for the system to maintain a homogeneous structure until the end of the curing process.

**Figure 9:**
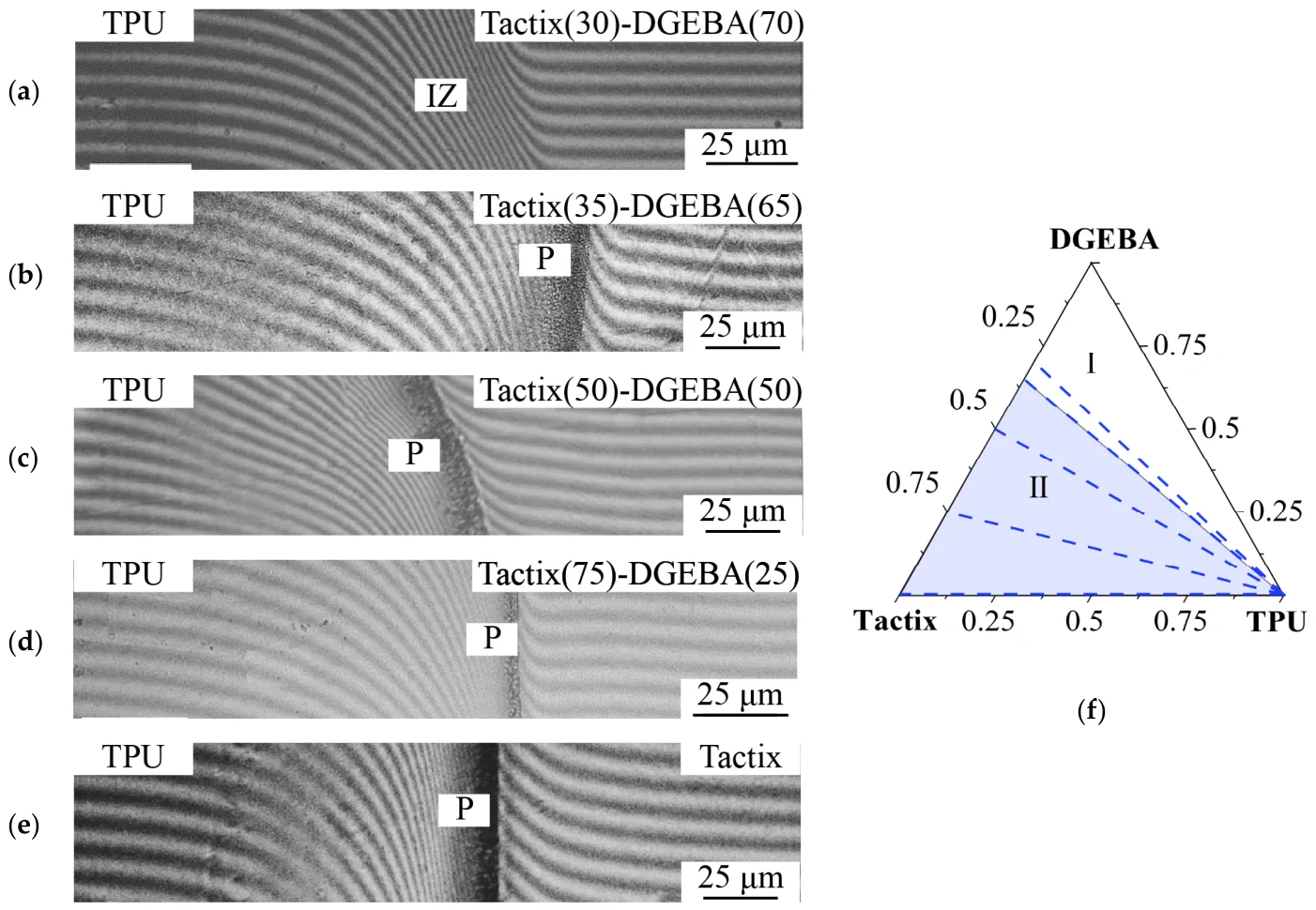
Interferograms of the interdiffusion zone during curing of the TPU–Tactix–DGEBA+DDS systems at 180 °C (data in parentheses indicate vol.%): (**a**) TPU–Tactix(30)–DGEBA(70), t = 127 min; (**b**) TPU–Tactix(35)–DGEBA(65), t = 55 min; (**c**) TPU–Tactix(50)–DGEBA(50), t = 43 min; (**d**) TPU–Tactix(75)–DGEBA(25), t = 26 min; (**e**) TPU–Tactix, at a temperature of 180 °C, t = 24 min; (**f**) Gibbs–Rosebohm triangle of the ternary system TPU–Tactix–DGEBA+DDS with the designated studied systems (blue dotted lines). Here, I is a homogeneous region; II is a heterogeneous region; IZ is the interdiffusion zone; P is the phase boundary. The blue dotted lines show the concentration cross-sections of the Gibbs triangle of the studied systems.

To predict the boundaries of the heterogeneous region in the ternary diagram of the TPU–Tactix–DGEBA system during DDS curing, quasi-binary mixtures were studied using optical interferometry. In these mixtures, Component 1 is TPU, and Component 2 is a reaction mixture of Tactix and DGEBA epoxy oligomers in various volume ratios with the addition of DDS according to stoichiometry (Supplementary Materials, Table S3). For clarity, some concentration ranges studied using quasi-binary systems are plotted on the Gibbs–Rosebohm triangle (Figure 9d).

Figure 9 shows interference pictures illustrating interdiffusion zones in the studied concentration ranges. It was found that at the initial stage of the curing reaction, all studied quasi-binary mixtures are characterized by complete mutual solubility of the components. Differences in phase transformations begin at later stages of curing. Phase behavior depends on the composition of the epoxy oligomer reaction mixture. Quasi-binary TPU compositions with the Tactix–DGEBA, containing ≥70 vol.% DGEBA in the oligomer mixture, maintain homogeneity until dense network structures are formed in the system (Figure 9a). That is, no signs of heterogeneity appear during the chemical curing reaction across the entire concentration range. This is likely due to the lower functionality of DGEBA and, consequently, the formation of a less dense epoxy network. In this case, the region of thermodynamic incompatibility of the system occurs after the gel point, when the macromolecular mobility necessary for the growth of heterogeneous structures by the nucleation mechanism is significantly limited.

The introduction of the trifunctional oligomer Tactix at an amount of ≥30 vol.% alters the nature of the interdiffusion region in the interferograms; specifically, the formation of a phase boundary characterizing the heterogeneity of the mixture is observed (Figure 9b–d). The higher functionality of the epoxy promotes the formation of a denser spatial network of chemical bonds. As a result, thermodynamic incompatibility is achieved before the gel point, when the system components retain sufficient mobility. With the increasing Tactix content, a decrease in the time required for phase boundary formation is also observed, due to the influence of the degree of conversion corresponding to the onset of phase separation.

The degree of conversion, or more precisely, the corresponding values of the interdiffusion coefficients at the onset of phase decomposition, has a fundamental influence on the sizes of the emerging phase structures. When phase decomposition initiates in the early stages of curing (low conversion), the diffusion coefficients remain high, facilitating intense mass transfer and the coarsening of phase structure domains. Conversely, as phase decomposition shifts toward the high conversion region, diffusion is limited, leading to a halt in structure formation in the early stages and the formation of a finely dispersed morphology.

**Figure 10:**
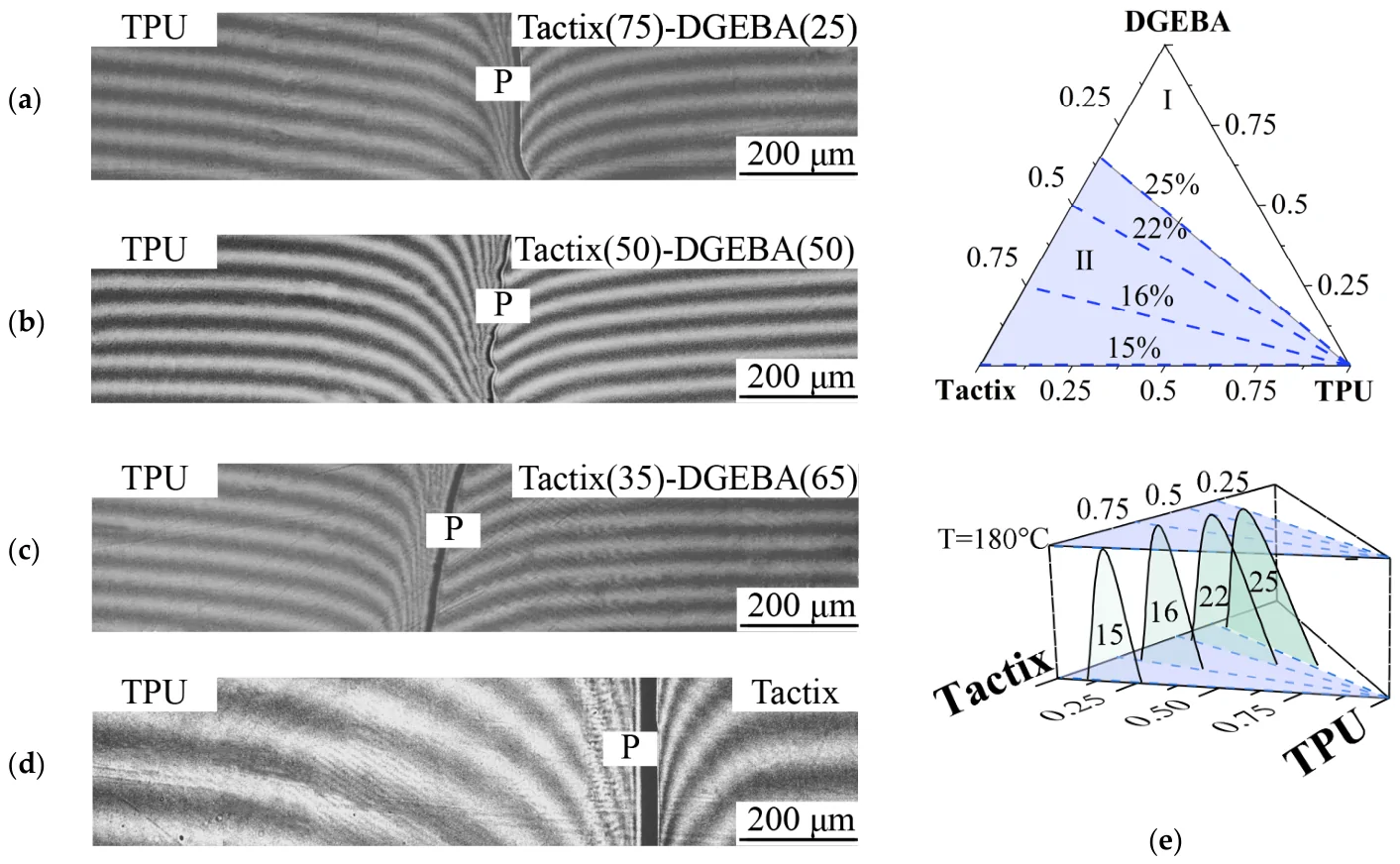
Interferograms of the interdiffusion zone of the TPU–Tactix–DGEBA+DDS systems at 180 °C (data in parentheses indicate vol.%): (**a**) TPU–Tactix(75)–DGEBA(25), α_pd_ = 25%; (**b**) TPU–Tactix(50)–DGEBA(50), α_pd_ = 22%; (**c**) TPU–Tactix(35)–DGEBA(65), α_pd_ = 16%; (**d**) TPU–Tactix, α_pd_ = 15%, at a temperature of 180 °C; and (**e**) Gibbs–Rosebohm triangle of the TPU–Tactix–DGEBA ternary system with schematically designated binodal curves of quasi-binary systems and the values of the corresponding conversion degrees when the binodal curves intersect with the isothermal concentration plane at a temperature of 180 °C. I is a homogeneous region; II is a heterogeneous region; P is the phase boundary. The blue dotted lines show the concentration cross-sections of the Gibbs triangle of the studied systems.

To assess the effect of the trifunctional Tactix oligomer on the degree of conversion at which phase decomposition is initiated, quasi-binary systems of TPU–adduct mixtures of Tactix–DGEBA were analyzed (Figure 10). The use of adducts allows for controlled variation in the conversion degree under curing conditions. It was established that an increase in the Tactix content leads to a shift in the onset of phase decomposition to the region of lower degrees of conversion (α_pd_), which is associated with the formation of a denser spatial network of chemical bonds.

### 3.3. Morphology of Cured Systems

To experimentally confirm the heterogeneous region identified by optical interferometry for the TPU–Tactix–DGEBA system, mixtures with compositions corresponding to a wide concentration range were prepared, encompassing both homo- and heterogeneous regions within the Gibbs–Rosebohm triangle. The resulting composites were cured at 180 °C, after which their morphology was analyzed.

**Figure 11:**
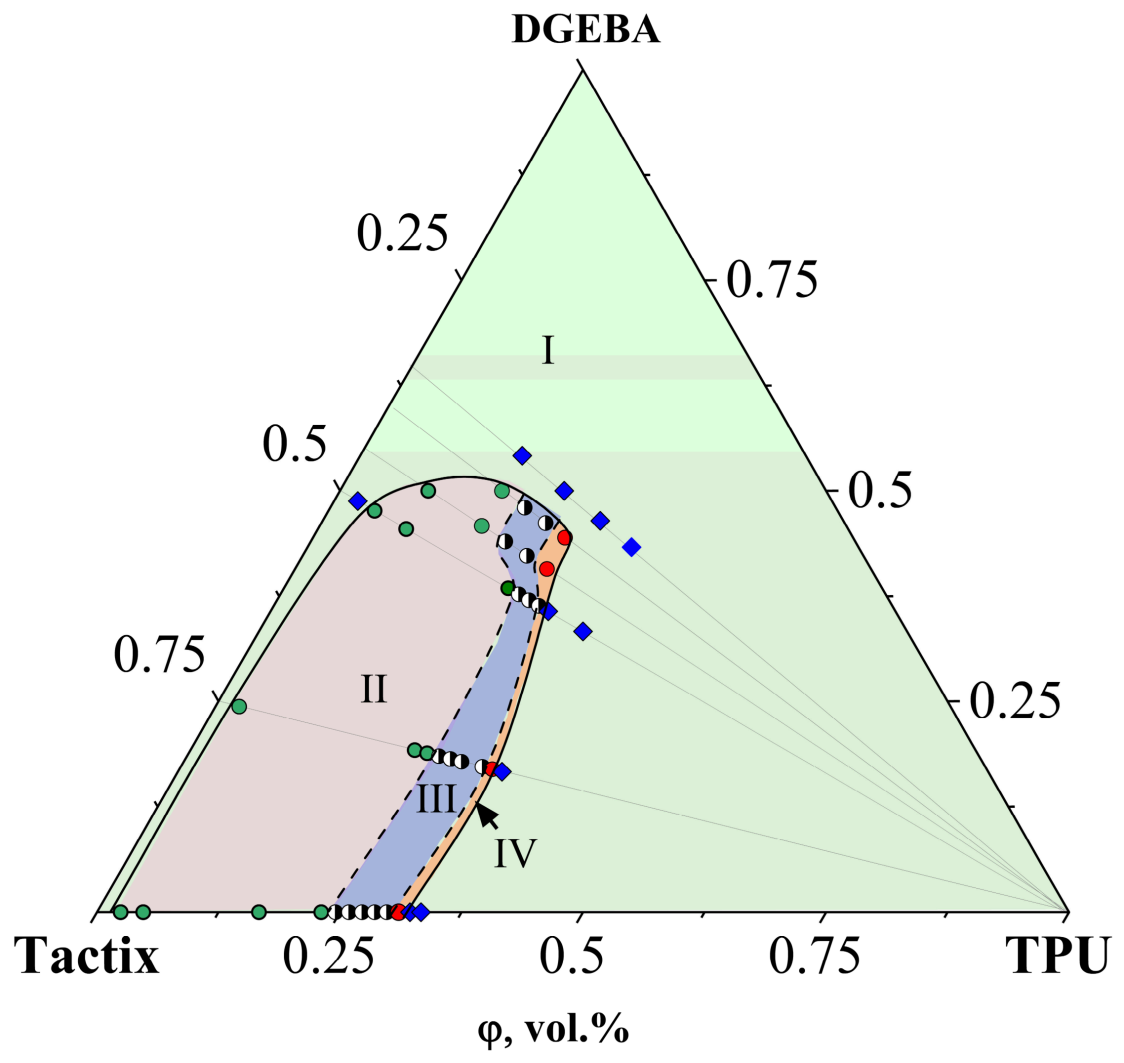
A ternary structural diagram showing the type of phase structure formed during curing of DDS at 180 °C depending on the composition of the TPU–Tactix–DGEBA system. I (◆) is a homogeneous region; II (●) is a region with a matrix–dispersion phase structure type; III (◑) is a region with an IPP-type phase structure; IV (●) is a region with an inverted phase structure. Solid and dashed lines indicate approximate transition regions.

**Figure 12:**
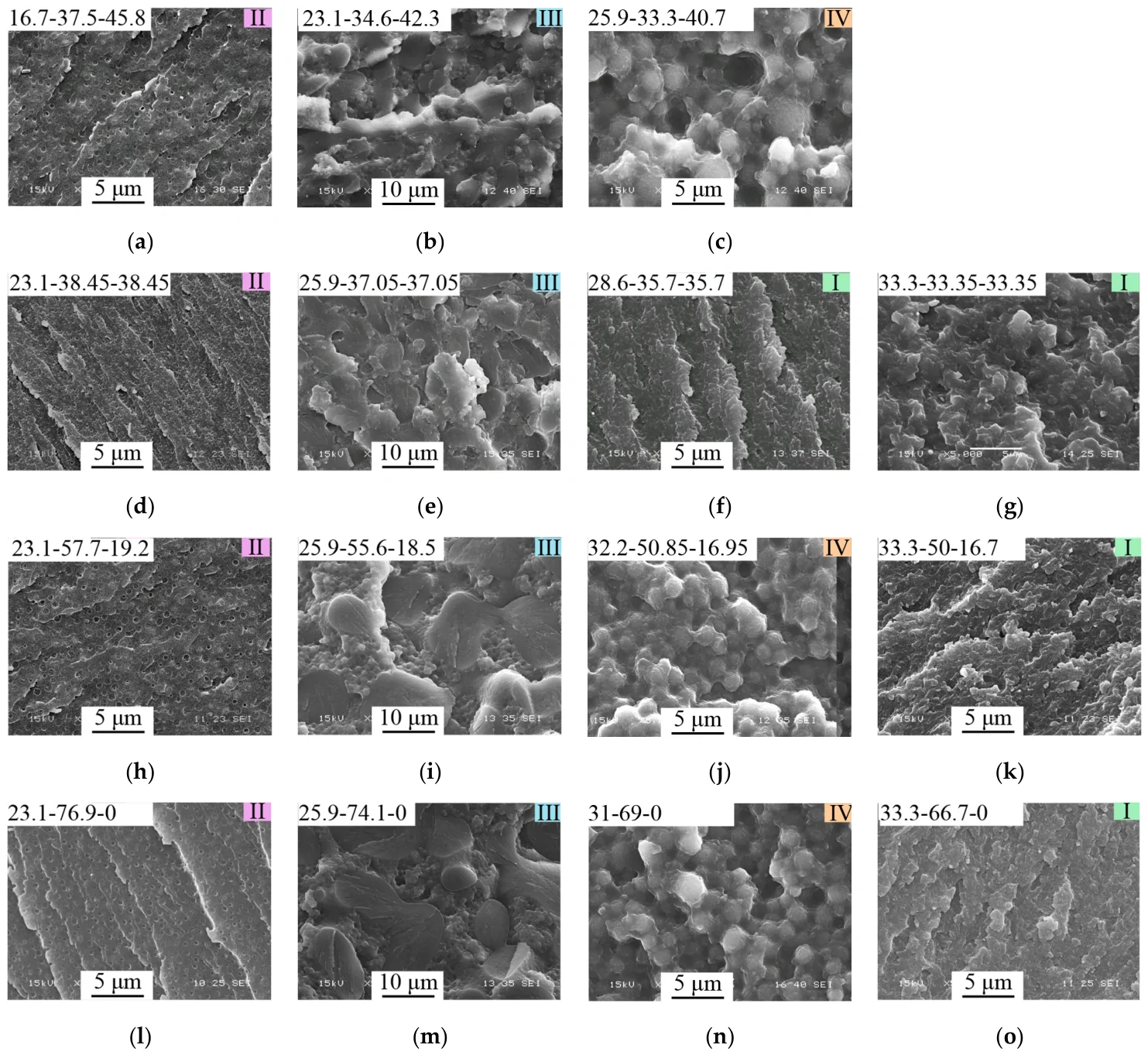
SEM images of the phase structure of cured TPU–Tactix–DGEBA+DDS systems at 180 °C. The component contents (vol.%), according to the component arrangement in the TPU–Tactix–DGEBA system name, are indicated in the images. The region of the diagram to which the structure belongs is also indicated: I—homogeneous region (**f**,**g**,**k**,**o**); II—matrix–dispersion region (**a**,**d**,**h**,**l**); III—region with IPP-type phase structure (**b**,**e**,**i**,**m**); IV—inverted phase region (**c**,**j**,**n**).

Based on SEM images, a structural diagram was constructed showing the type of phase structure that formed depending on the mixture composition (Figure 11). It should be noted that this diagram is not a phase diagram in the classical sense, as one of the key characteristics of phase diagrams is their equilibrium nature. In this case, the positions of the binodal boundaries correspond to the compositions of the coexisting phases, fixed at different degrees of conversion during curing, thus showing the nonequilibrium state of the system. Thus, the presented structural diagram can be considered a concentration map of the final phase structure of the composite formed at a given curing temperature (180 °C) for the TPU–Tactix–DGEBA system. The resulting structural diagram is not applicable to other curing modes, since changing the curing temperature can alter the reaction kinetics and the mobility of the components that influence the formation of the phase structure. The areas highlighted in it are confirmed by the results of direct morphology studies. The ternary structural diagram (Figure 11) shows the areas of different phase structures in the TPU–Tactix–DGEBA system, and Figure 12 shows the corresponding SEM images. SEM images are shown at fixed magnifications for each type of phase structure, which ensures a correct comparison of the sizes of structural elements (5000× for the matrix–dispersion, phase inversion, and homogeneous region morphologies; 2500× for the IPP).

Region I (Figure 11) corresponds to a homogeneous structure, in which the material properties are determined by average values, taking into account the mass contribution of the components. It should be noted that, despite the potential thermodynamic incompatibility of the components in the system, at a high TPU content (≥30 vol.%), phase separation does not occur. Phase separation can be suppressed due to an increase in the system viscosity and a decrease in the interdiffusion coefficients. However, TPU, as shown above (Figure 4), is characterized by better diffusion mobility compared to other components of the system. Therefore, the absence of phase separation in systems with a high TPU content is explained by a decrease in the reactivity of the epoxy oligomer due to its dissolution in the continuous thermoplastic matrix. Thus, the required degree of epoxy conversion for phase separation in the system is not achieved.

Region II (Figure 11) is characterized by a heterogeneous matrix–dispersion morphology, where the dispersed phase is enriched in TPU, and the matrix is a mixture of Tactix–DGEBA epoxy polymers. This region II has a width of ~25 vol.%, while the phase inversion region (IV), where the matrix is enriched in TPU and the dispersion is enriched in epoxy resin, has a width of ~2 vol.%. Region III represents a structure of interpenetrating phases (IPPs), which is optimal for increasing the crack resistance of the material [7,32], and the width of the IPP region is ~8 vol.%. Thus, by changing the TPU concentration in a small range (within 10 vol.%), it is possible to select the morphology among four types of structures (homogeneous and three heterogeneous types) depending on the operational tasks for which the composite material is developed.

**Figure 13:**
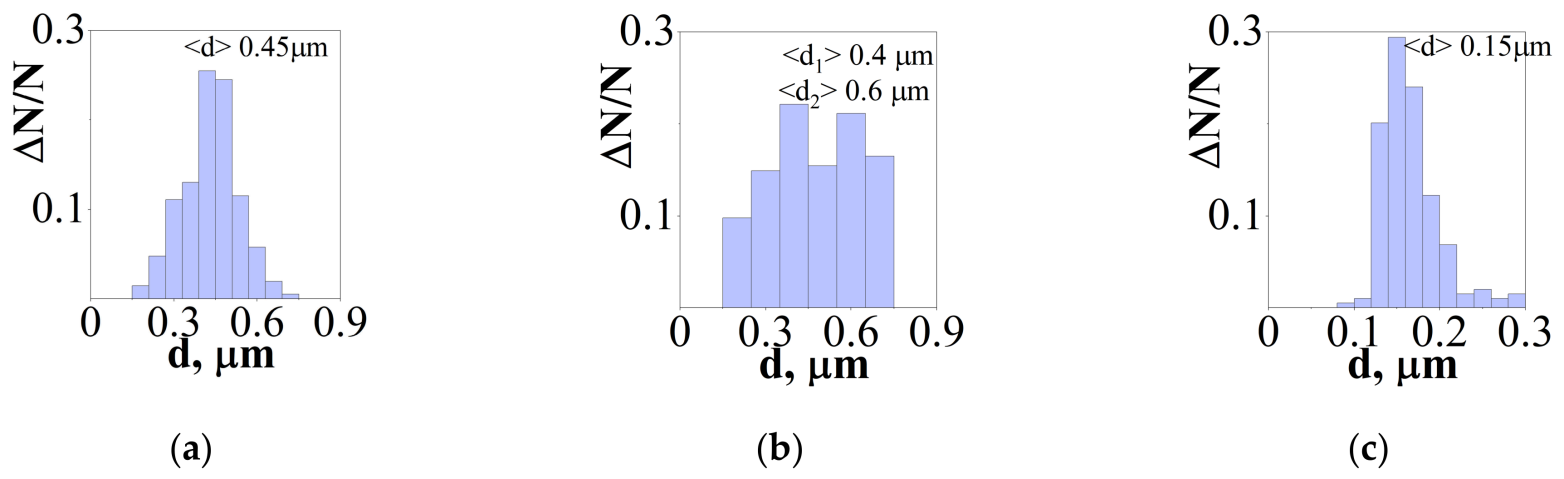
Particle size distribution of the dispersed phase in the cured TPU–Tactix–DGEBA+DDS system with a TPU content of 23.1 vol.%, and with a Tactix–DGEBA content (vol.%): (**a**) 76.9–0; (**b**) 57.7–19.2; (**c**) 38.45–38.45. The average particle diameter (μm) is shown in the figures.

Differences between the degrees of conversion of phase decomposition initiation and structure fixation, as well as the values of the interdiffusion coefficients of the components at these stages, determine the characteristic size of the forming phase structures. This can be most clearly assessed using a composition with a matrix–dispersion phase structure with a fixed TPU content (23.1 vol.%) and a varying composition of the Tactix–DGEBA epoxy mixture. The particle sizes of the dispersed phase were estimated manually using the FIJI software package [33]. For each concentration, more than 200 particles selected from three independent SEM fields of view were analyzed, ensuring the reliability of the averaged values. The particle size distributions of the dispersed phase for different mixture compositions are shown in Figure 13.

The particle size distribution of the dispersed phase for the TPU (23.1 vol.%)–Tactix–DGEBA+DDS mixture with a Tactix–DGEBA content of 76.9–0 showed a maximum in the 0.45 µm range. The mean particle diameter was 0.43 µm (standard deviation ± 0.1 µm), and the size range was 0.159–0.697 µm. For the system with a Tactix–DGEBA content of 57.7–19.2, the mean particle diameter was 0.477 ± 0.167 µm. The particle size distribution was bimodal, with maxima in the 0.4 and 0.6 µm ranges. For the system with a Tactix–DGEBA content of 38.45–38.45, the particle size distribution showed a maximum in the 0.15 µm range. The mean particle diameter was 0.172 ± 0.037 µm, and the size range was 0.080–0.299 µm.

In the literature [34,35,36], it is noted that the theoretical (ideal) degree of conversion at the gel point can be calculated using the Flory–Stockmayer equation (Equation (4)). Functionality for the Tactix–DGEBA mixed compositions was determined using Equation (5) (Table 2) [37]. The complete calculation is provided in the Supplementary Materials.

(4) $$\alpha_{gel}^{theory}={\left[\left(f-1\right)\left(g-1\right)\right]}^{-\frac{1}{2}}$$

(5) $$f_{Tactix-DGEBA}=\frac{{\;\;\;\;f}_{Tactix}^{2}N_{Tactix}+f_{DGEBA}^{2}N_{DGEBA}}{{f_{Tactix}N}_{Tactix}+{f_{DGEBA}N}_{DGEBA}}$$

Here, f is the functionality of the epoxy resin, g is the functionality of the hardener, and N is the number of moles of epoxide.

The term “theoretical” refers to the equal reactivity of functional groups of the same type and the absence of substitution effects. (The functional groups remaining in the partially reacted DGEBA retain their original reactivity). Furthermore, the Flory–Stockmeyer model does not take into account molecular weight distribution, possible differences in the reactivity of functional groups, or heterogeneity in the curing reaction. Experimental determination of the gel point using a rheological method was not performed in this study.

The obtained values of the average size of the dispersed phase, the degree of conversion at the onset of phase decomposition (α_pd_) (Figure 10), and the theoretical degree of gelation ($\alpha_{gel}^{theory}$) for the TPU–Tactix–DGEBA system are presented in Table 2. The reduction in the time required to reach the gel point as the proportion of Tactix increases is consistent with a decrease in the theoretically calculated degree of conversion (Supplementary Materials, Figure S3).

The obtained particle size distributions indicate that in TPU samples with the Tactix–DGEBA epoxy mixture in the ratio (75–25), an increase in the average size of the TPU-enriched dispersed phase is observed compared to the Tactix–DGEBA (100–0) composition. It was also established that the degrees of conversion corresponding to the onset of phase decomposition for these systems are comparable (15 and 16%), indicating similar conditions for the initiation of phase decomposition. In this case, the increase in the size of the dispersed phase is due to the higher diffusion mobility of DGEBA (Section 3.1) and its lower functionality, which is reflected in a shift in the gel point to the region of higher degrees of conversion ($\alpha_{gel}^{theory}$ = 43.7%). As a result, the formation time of heterogeneous structures under conditions of good diffusion mobility increases.

In the Tactix–DGEBA (50–50) system, a decrease in the average particle size to 0.15 μm is observed. Despite the high initial diffusion mobility of DGEBA, phase decomposition begins at a higher conversion rate (22%), resulting in a shorter time interval to gelation. The necessary conditions for obtaining the finest dispersed phase structures in the cured system are phase separation at high degrees of conversion and gelation at the lowest possible degrees of conversion. However, the observed decrease in the size of the dispersed phase cannot be explained solely by a reduction in the time interval, since the differences between the determined theoretical values of the conversion degree at the gel point and the conversion degree at the beginning of phase decomposition for the Tactix–DGEBA compositions (100–0) and (50–50) are insignificant (25.8 and 25%).

This effect is likely due to the dependence of diffusion coefficients on the conversion degree, which limits mass transfer in the case of a later onset of phase decomposition [38]. Thus, the size of the final morphology is related not only to the size of the interval between the onset of phase decomposition and gelation, but also to the kinetic decrease in diffusion mobility during curing.

It should be noted that previous fundamental research into phase equilibria in thermoplastic-modified reactive systems has enabled the development of approaches for predicting the type and parameters of phase structures based on data on phase equilibria and diffusion coefficients of the initial systems, as well as their evolution during the curing reaction [23]. This created the basis for the transition from fundamental work to solving applied problems. The structural diagram obtained in this study largely reflects the applied needs of developers of modern composite materials with the required set of performance characteristics and technologists working on the production of materials with a given phase structure.

## 4. Conclusions

In this study, the phase equilibria, diffusion characteristics, and structure formation kinetics in the ternary system thermoplastic polyurethane (TPU)–trifunctional epoxy oligomer (Tactix)–difunctional epoxy oligomer (DGEBA) during curing with the aromatic amine DDS were investigated using an integrated approach combining optical interferometry, FTIR spectroscopy, differential scanning calorimetry, and scanning electron microscopy.

Optical interferometry was used to establish the thermodynamic compatibility of the components in their initial state. All three basic binary systems (TPU–DGEBA, TPU–Tactix, and Tactix–DGEBA) are characterized by complete mutual solubility of the components across the entire concentration range and a wide temperature range (80–190 °C). Experimental isoconcentration kinetic dependences confirm the purely diffusion mechanism of mixing the components and the absence of a chemical interaction between them before the introduction of the hardener.

FTIR spectroscopy showed that the TPU–Tactix blend is stable and does not undergo chemical reactions prior to curing. However, TPU undergoes conformational changes when mixed with epoxy resin, associated with the disruption of its own hydrogen bonds (disappearance of the bound carbonyl peak at 1704 cm^−1^). This can lead to the formation of new weak intermolecular contacts such as –N–H O=C– between the polar groups of the polyurethane and the hydroxyl groups/oxygen of the epoxy rings.

The calculated interdiffusion coefficients range from 10^−6^ to 10^−7^ (cm^2^/s), indicating high component mobility. The macromolecular mobility of the difunctional DGEBA oligomer (E_a_ = 36.6 kJ/mol) was found to exceed that of the trifunctional Tactix (E_a_ = 46.5 kJ/mol). A significant drop in diffusion coefficients was observed for the TPU–DGEBA system at 130 °C, due to the crystallization of the rigid TPU segments.

The topology of phase transformations on the Gibbs–Roozeboom triangle was determined by studying the quasi-binary cross-sections of the ternary system during curing. It was shown that compositions with a high DGEBA content (over 70 vol.%) maintain homogeneity throughout the reaction due to the formation of a rarefied spatial network, shifting the onset of phase separation beyond the gel point. Systems with a high TPU content (over 30 vol.%) also remain homogeneous due to kinetic inhibition (with a high viscosity of the medium). The addition of Tactix (over 30 vol.%) accelerates the achievement of thermodynamic incompatibility and shifts the onset of phase separation to the region of earlier conversions (from 22 to 15%).

Using SEM, a detailed concentration map (structural diagram) of the final morphologies of cured composites at 180 °C was constructed. Four topological regions were experimentally recorded and described: a homogeneous structure (I), a heterogeneous matrix–dispersion structure with TPU-enriched phases (II), a region of interpenetrating phases (III), and an inverted phase region with a continuous TPU-enriched matrix (IV).

It has been shown that the average particle diameter of the dispersed phase correlates strongly with the conversion degree at the onset of phase decomposition and the position of the theoretical gel point calculated using the Flory–Stockmayer theory. The maximum particle size (up to 0.6 μm) is achieved with the Tactix–DGEBA (75–25) combination due to the high diffusion mobility of DGEBA and the wide time interval before gelation. A shift in decomposition to later stages (22% conversion for the 50–50 mixture) leads to the formation of a highly dispersed structure with a particle size of approximately 0.15 μm due to a decrease in the diffusion coefficients in the reacting system. The developed interference–microscopic approach and the constructed structural diagram of the ternary TPU–Tactix–DGEBA system open up the technological possibility of precise control of the phase architecture of epoxy compounds, including the formation of the IPP-type structure within a narrow concentration range (10 vol.%) without changing the chemical nature of the initial components.

## Figures and Tables

**Table 1:** Characteristic absorption band ranges for TPU and Tactix.

Component	Range, cm^−1^	Absorption Band
TPU	3400–3300	N–H stretching vibrations
Tactix, TPU	3100–2800	C–H stretching vibrations (including aromatic C–H)
TPU	1750–1700	C=O stretching vibrations of the carbonyl group
Tactix	1630–1400	C=C stretching vibrations in the aromatic ring
TPU	1597	aromatic ring stretching vibrations of diisocyanate
TPU	1530	Amide II
Tactix	1300–1000	Ar–O–C asymmetric stretching vibrations of aryl-ether bonds
TPU	1250–1200	C–O stretching vibrations in the urethane group
TPU	1100–1050	C–O–C stretching vibrations of the polyether soft segment
Tactix	920–900	stretching vibrations of the epoxy ring—a marker for resin curing
Tactix	840–830	Out-of-plane C–H bending vibrations of the aromatic ring

**Table 2:** Average size of the dispersed phase, degree of conversion at the onset of phase decomposition and theoretical degree of gelation for the TPU–Tactix–DGEBA system with a TPU content of 23.1 vol.% (30 parts by mass).

Tactix–DGEBA Ratio in the Mixture	f_epoxy_	N_Tactix_/N_DGEBA_	Content of Tactix–DGEBA in the Mixture, vol.%	α_pd_, %	<d>, µm	$\left(\alpha_{gel}^{theory}\right)$, %
100–0	3	–	76.9–0	15	0.45	40.8
75–25	2.54	0.104/0.133	57.7–19.2	16	0.4; 0.6	43
50–50	2.78	0.156/0.066	38.45–38.45	22	0.15	47
0–100	2	–	0–76.9	–*	–*	57.7

* α_pd_ and <d> are not reported for the Tactix–DGEBA (0–100) system because the system is homogeneous under the experimental conditions.

## Data Availability

Data are available upon request to the authors.

## Supplementary Materials

The following supporting information can be downloaded at https://www.mdpi.com/article/10.3390/polym18202605/s1. Table S1. Main characteristics of the components of the polymer systems under study; Table S2. Systems investigated by optical interferometry; Table S3. Systems investigated by optical interferometry; Table S4. Compositions of the studied systems and corresponding SEM images of systems cured at 180 °C. Roman numerals indicate the region of the diagram to which the structure belongs: I—homogeneous region, II—region with a matrix–dispersion phase structure, III—region with IPP-type phase structure, IV—region with inverted phase structure. For compositions 32.5–100–0 (III), 45–100–0 (IV) and 40–75–25 (III), SEM images of two independent samples are shown. Figure S1. DSC thermogram of the initial components of TPU, Tactix and their mixture TPU (33.3 vol.%)—Tactix (66.7 vol.%), second heating; Figure S2. FTIR spectra of the mixture TPU (33.3 vol.%)—Tactix (66.7 vol.%) after sorption tests using the desiccator method at various humidities (p/p_s_ = 0, 0.47 (room conditions), 0.6, and 1); Figure S3. Dependence of viscosity on curing time for DDS-based systems: DGEBA, Tactix, and their mixture, Tactix(75)–DGEBA(25).
