# Supplementary material for: Phase Equilibria and Morphology of the TPU–Tactix–DGEBA Ternary System: Interference Approach and SEM Analysis
Source: Polymers (Basel). 2026 Oct 21;18(20):2605. doi: 10.3390/polym18202605 (PMC13709124; doi:10.3390/polym18202605)
Supplement: Supplementary file 1 [file polymers-18-02605-s001.zip › polymers-4587876-supplementary.pdf]

polymers

MDPI

Article

Phase Equilibria and Morphology of the TPU–Tactix–DGEBA Ternary System: Interference Approach and SEM Analysis

Artem D. Ponomarenko, Irina O. Plyusnina, Uliana V. Nikulova and Aleksey V. Shapagin*

Frumkin Institute of Physical Chemistry and Electrochemistry, Russian Academy of Sciences (IPCE RAS), 31-4, Leninsky prospect, 119071 Moscow, Russia; ponomarenhik@gmail.com (A.D.P.); irinaplyusninar@yandex.ru (I.O.P.); ulianan@rambler.ru (U.V.N.)

* Correspondence: shapagin@mail.ru

Supplementary materials

Table S1. Main characteristics of the components of the polymer systems under study.

Component	ρ, g/cm³	Stoichiometric ratio of DDS to resin, parts by mass	EEW/AHEW
TPU	1.2	–	–
DGEBA	1.2	100:33	188.5
Tactix	1.2	100:38.8	160
DDS	–	–	62

Calculation of the stoichiometric ratio of DDS to resin:

$$X_{eq.DDS} = \frac{\mathrm{AHEW}_{DDS}}{EEW_{Epoxy}} \times 100 \quad (1)$$

Calculation of the DDS content (mass parts) for obtaining epoxy oligomer adducts with a given degree of conversion:

$$\alpha = \frac{m_{DDS}/\mathrm{AHEW}_{DDS}}{m_{Tactix}/EEW_{Tactix} + m_{DGEBA}/EEW_{DGEBA}} \times 100\% \quad (2)$$

$$m_{DDS} = \frac{\alpha}{100}\left(m_{Tactix}/EEW_{Tactix} + m_{DGEBA}/EEW_{DGEBA}\right) \times \mathrm{AHEW}_{DDS} \quad (3)$$

Table S2. Systems investigated by optical interferometry.

Investigation of curing with epoxy oligomer adducts by optical interferometry at 180°C				
Component 1	Component 2			
TPU, parts by mass	Tactix, parts by mass	DGEBA, parts by mass	DDS, parts by mass	a, %
100	100	0	5.81	15
	75	25	5.96	16
	50	50	7.88	22
	35	65	8.73	25

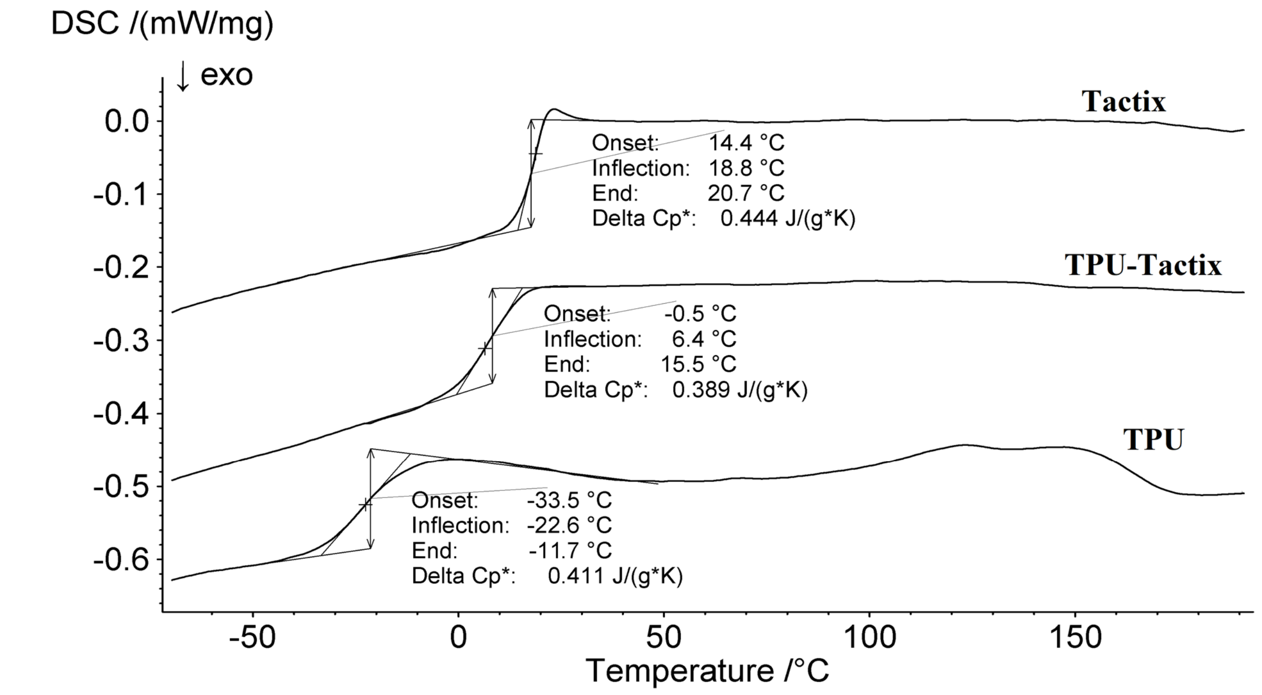

Figure S1. DSC thermogram of the initial components of TPU, Tactix and their mixture TPU (33.3 vol.%) – Tactix (66.7 vol.%), second heating.

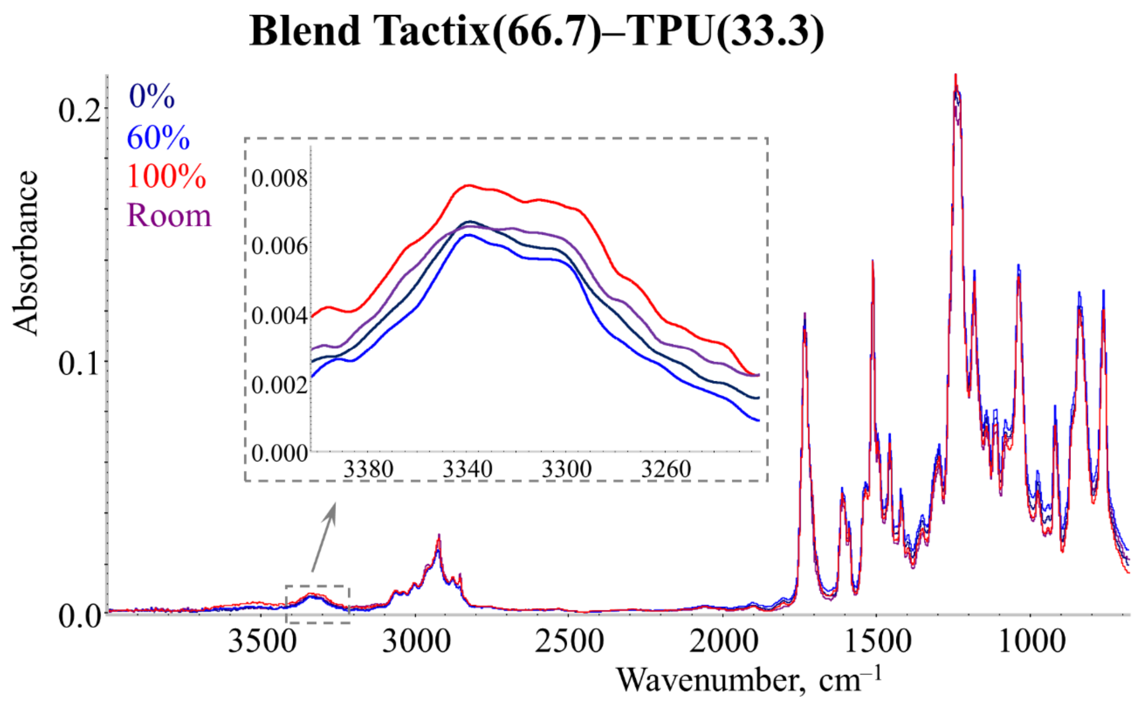

Figure S2. FTIR spectra of the mixture TPU (33.3 vol.%) – Tactix (66.7 vol.%) after sorption tests using the desiccator method at various humidities (p/ps = 0, 0.47 (room conditions), 0.6, and 1).

Table S3. Systems investigated by optical interferometry.

**Investigation of curing by optical interferometry at 180°C**			
**TPU, parts by mass**	**Tactix, parts by mass**	**DGEBA, parts by mass**	**DDS, parts by mass**
100	100	0	38.8
	75	25	37.3
	50	50	35.8
	40	60	35.2
	35	65	34.9
	30	70	34.6

Complete calculation of the theoretical (ideal) conversion degree at the gel point:

$$f = \Sigma(f^2N) / \Sigma(fN)$$

Composition Tactix/DGEBA = 50/50

Tactix: f = 3; M = 480 g/mol; DGEBA: f = 2; M ≈ 377 g/mol

$$N_{Tactix} = 50 / 480 = 0.1042 \text{ mol}$$

$$N_{DGEBA} = 50 / 377 = 0.1326 \text{ mol}$$

$$f = [3^2 \times 0.1042 + 2^2 \times 0.1326] / [3 \times 0.1042 + 2 \times 0.1326]$$

$$f = 1.4680 / 0.5778 = 2.54$$

$$\mathbf{f \approx 2.54}$$

Composition Tactix/DGEBA = 75/25

$$N_{Tactix} = 75 / 480 = 0.1563 \text{ mol}$$

$$N_{DGEBA} = 25 / 377 = 0.0663 \text{ mol}$$

$$f = [3^2 \times 0.1563 + 2^2 \times 0.0663] / [3 \times 0.1563 + 2 \times 0.0663]$$

$$f = 1.6715 / 0.6014 = 2.78$$

$$\mathbf{f \approx 2.78}$$

$$r = (m_{DDS} / AHEW_{DDS}) / [m_{Tactix} / EEW_{Tactix} + m_{DGEBA} / EEW_{DGEBA}]$$

AHEW(DDS) = 62 g/eq.

50/50 + 35.8 DDS:

$$r = (35.8 / 62) / [(50 / 160) + (50 / 188.5)]$$

$$r = 0.5774 / 0.5778 = 0.999 \approx 1.00$$

75/25 + 37.3 DDS:

$$r = (37.3 / 62) / [(75 / 160) + (25 / 188.5)]$$

$$r = 0.6016 / 0.6014 = 1.0 \approx 1.0$$

**Tactix/DGEBA**	**DDS, parts by mass**	**f**	**r**	α_{gel}^{theory}
50/50	35.8	2.54	1.0	**47**
75/25	37.3	2.78	1.0	**43**

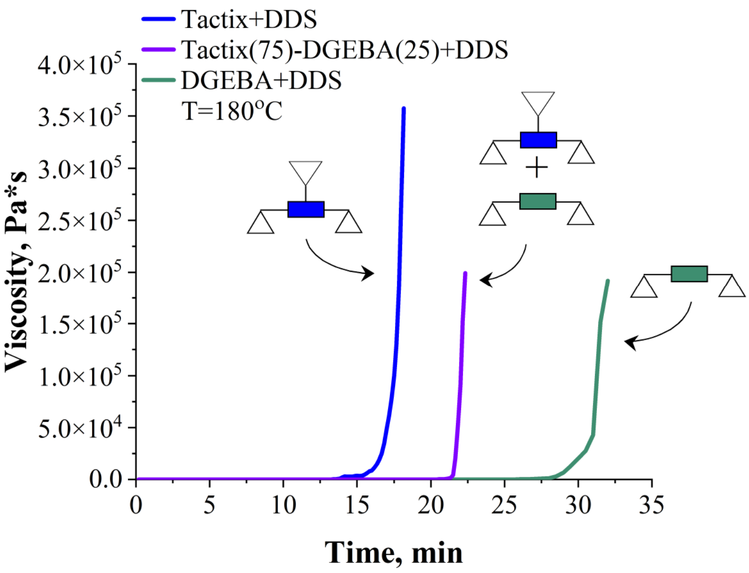

Figure S3. Dependence of viscosity on curing time for DDS-based systems: DGEBA, Tactix, and their mixture, Tactix(75)–DGEBA(25).

Table S4. Compositions of the studied systems and corresponding SEM images of systems cured at 180°C. Roman numerals indicate the region of the diagram to which the structure belongs: I – homogeneous region, II – region with a matrix–dispersion phase structure, III – region with IPP-type phase structure, IV – region with inverted phase structure. For compositions 32.5–100–0 (III), 45–100–0 (IV) and 40–75–25 (III), SEM images of two independent samples are shown.

**TPU, vol.%**	**Tactix, vol.%**	**DGEBA, vol.%**	**TPU–Tactix–DGEBA, parts by mass**	**DDS, parts by mass relative to the resin component**	**CЭM-images**
2.4	97.6	0.0	2.5–100–0 (II)	38.8	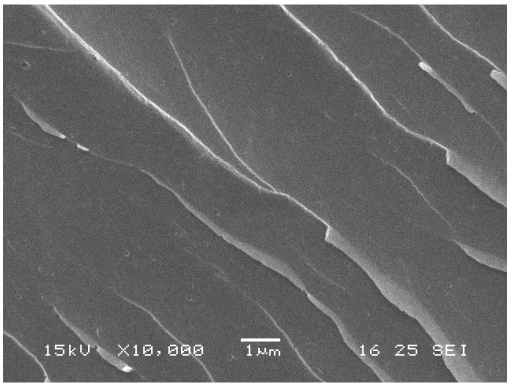

4.8	95.2	0.0	5–100–0 (II)	38.8	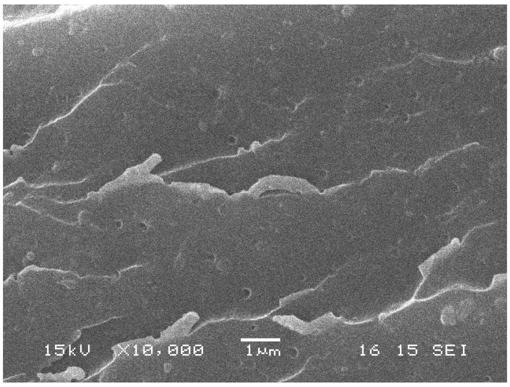
16.7	83.3	0.0	20–100–0 (II)	38.8	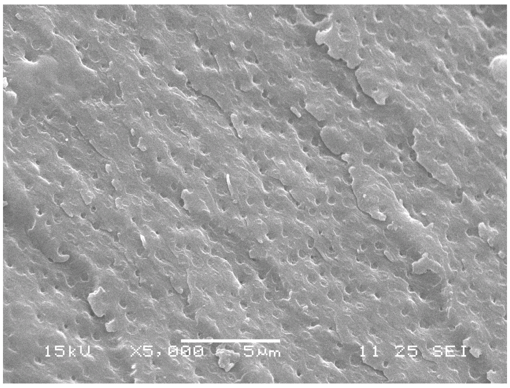
23.1	76.9	0.0	30–100–0 (II)	38.8	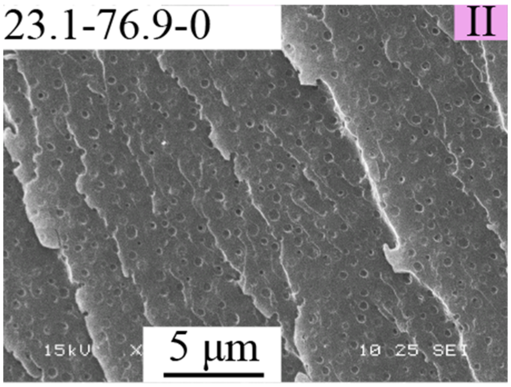
24.5	75.5	0.0	32.5–100–0 (III)	38.8	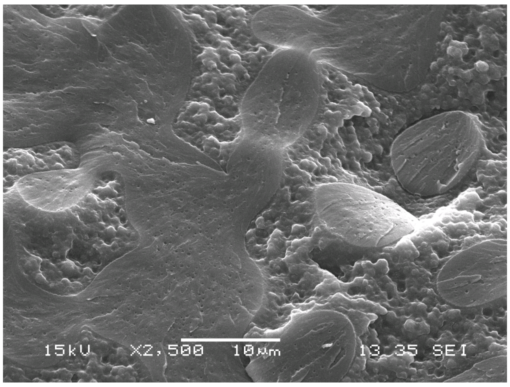

					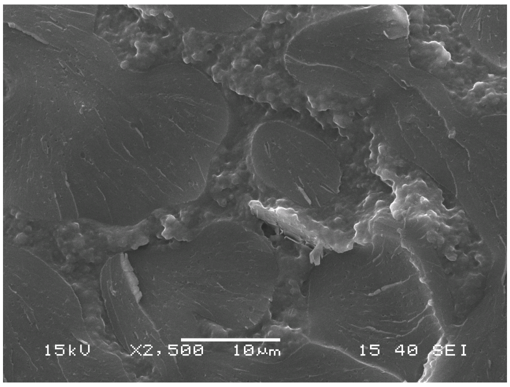
25.9	74.1	0.0	35–100–0<br>(III)	38.8	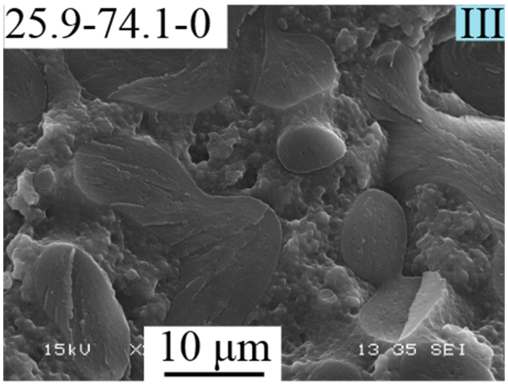
27.3	72.7	0.0	37.5–100–0<br>(III)	38.8	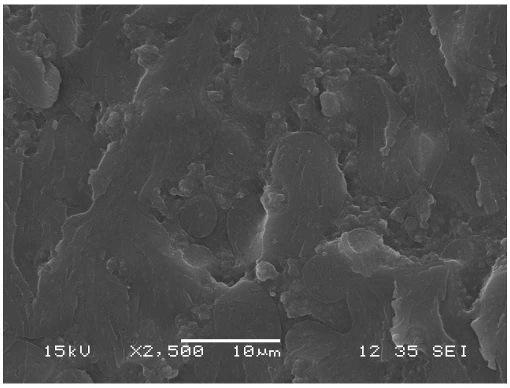
28.6	71.4	0.0	40–100–0<br>(III)	38.8	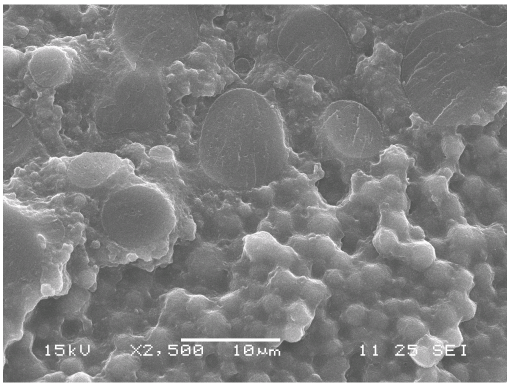

29.8	70.2	0.0	42.5–100–0<br>(III)	38.8	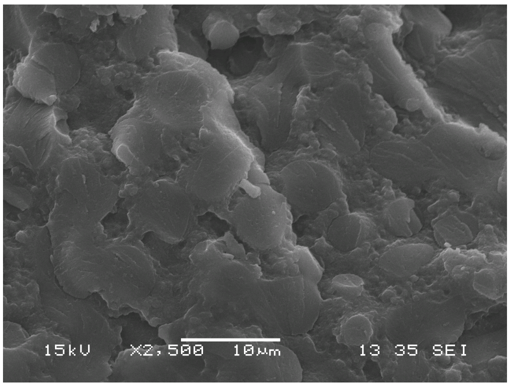
31.0	69.0	0.0	45–100–0<br>(IV)	38.8	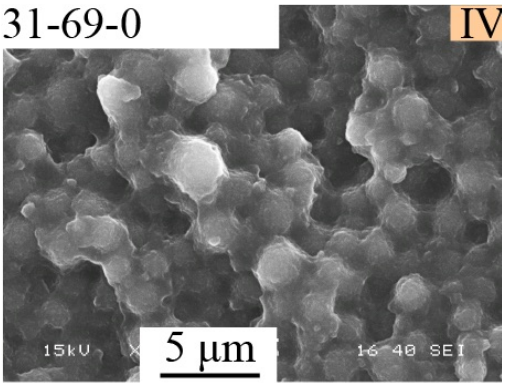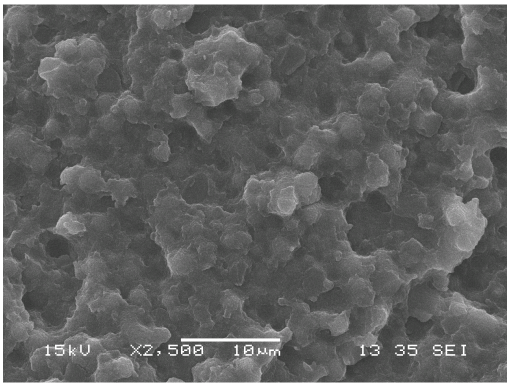
32.2	67.8	0.0	47.5–100–0<br>(I)	38.8	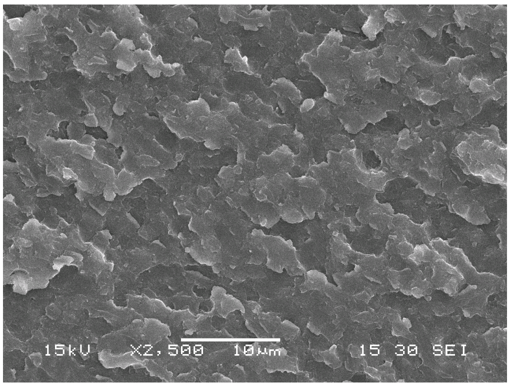

33.3	66.7	0.0	50–100–0<br>(I)	38.8	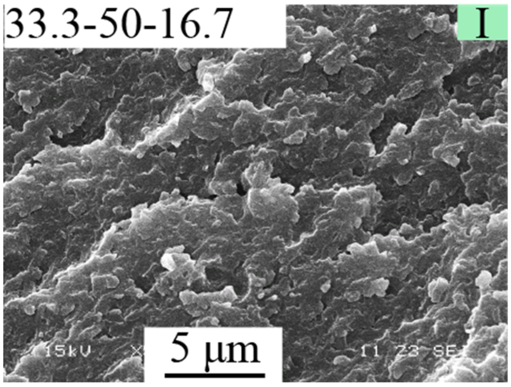
2.4	73.2	24.4	2.5–75–25<br>(II)	37.3	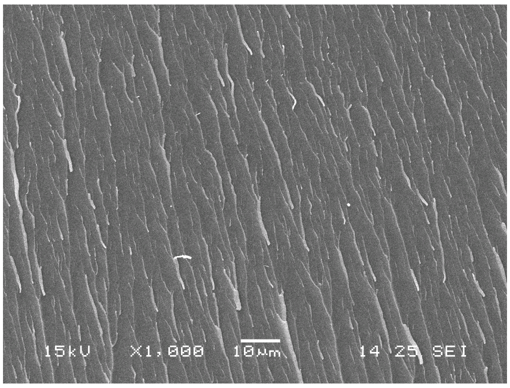
23.1	57.7	19.2	30–75–25<br>(II)	37.3	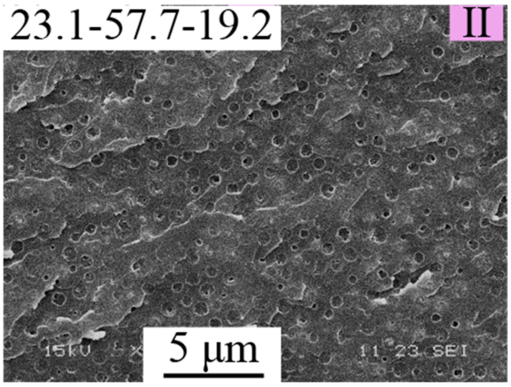
24.5	56.6	18.9	32.5–75–25<br>(II)	37.3	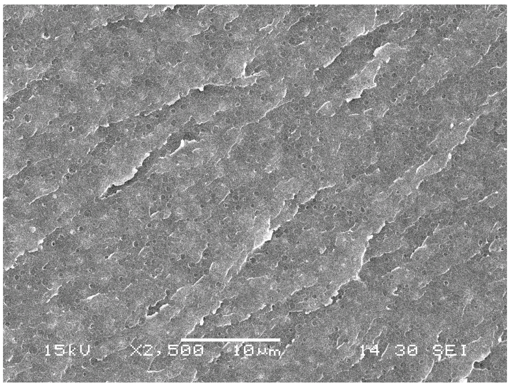

25.9	55.6	18.5	35–75–25<br>(III)	37.3	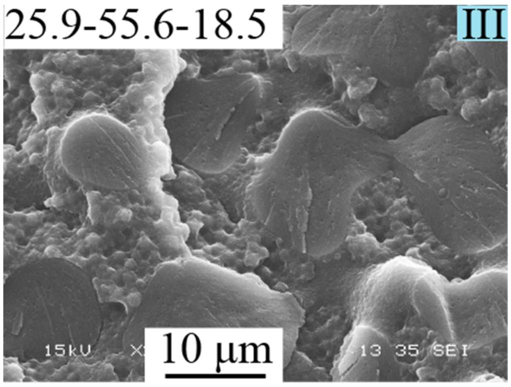
27.3	54.5	18.2	37.5–75–25<br>(III)	37.3	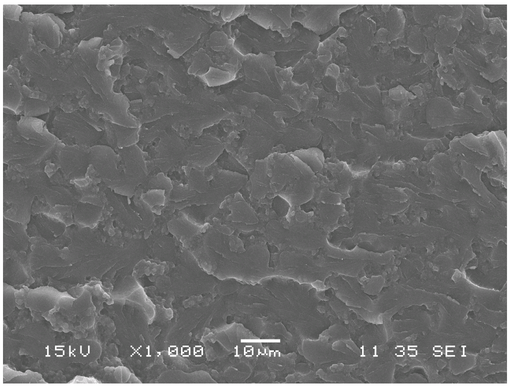
28.6	53.6	17.9	40–75–25<br>(III)	37.3	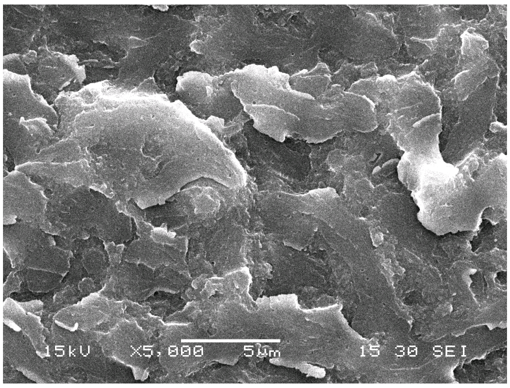 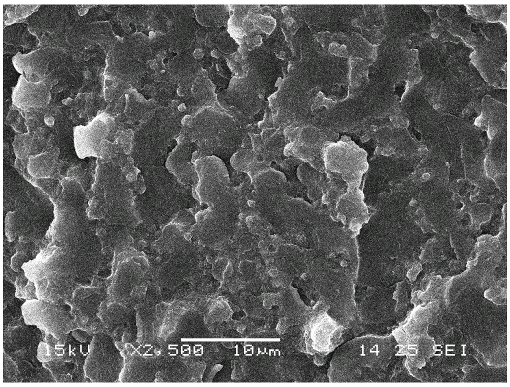

31.0	51.7	17.2	45–75–25<br>(III)	37.3	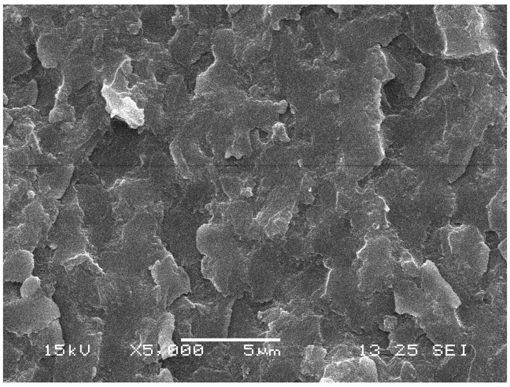
32.2	50.8	16.9	47.5–75–25<br>(IV)	37.3	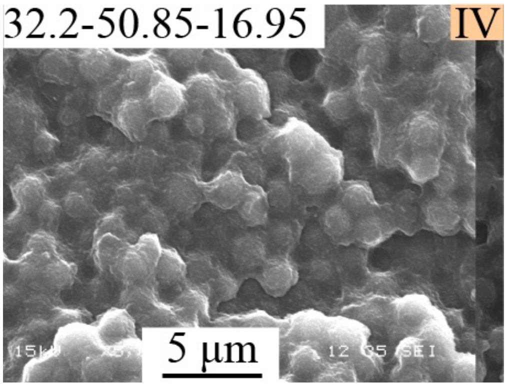
33.3	50.0	16.7	50–75–25<br>(I)	37.3	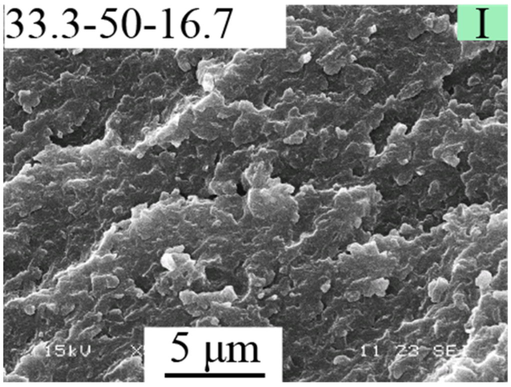
2.4	48.8	48.8	2.5–50–50<br>(I)	35.8	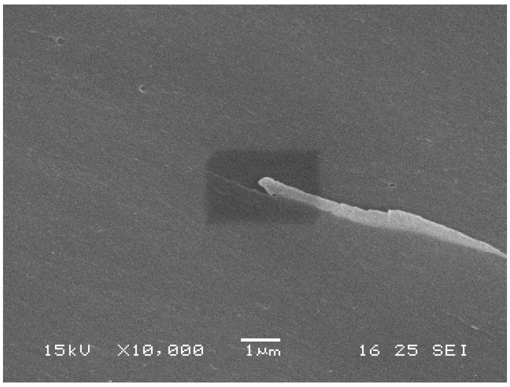

9.1	45.5	45.5	10–50–50<br>(II)	35.8	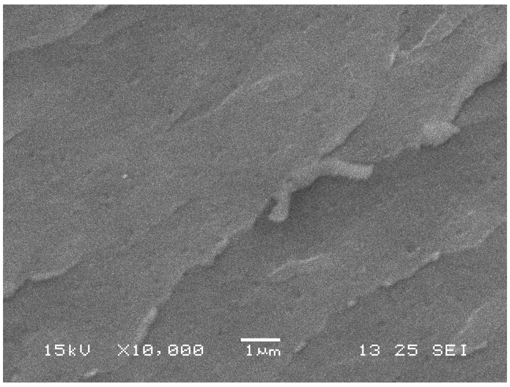
23.1	38.5	38.5	30–50–50<br>(II)	35.8	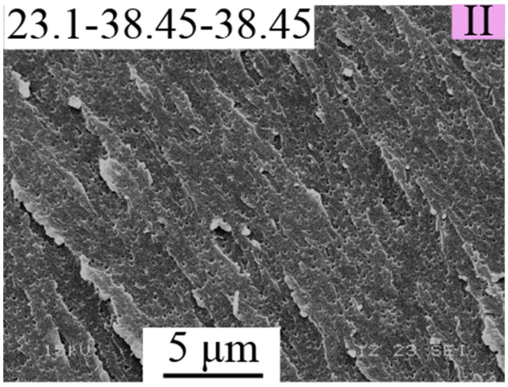
24.5	37.7	37.7	32.5–50–50<br>(III)	35.8	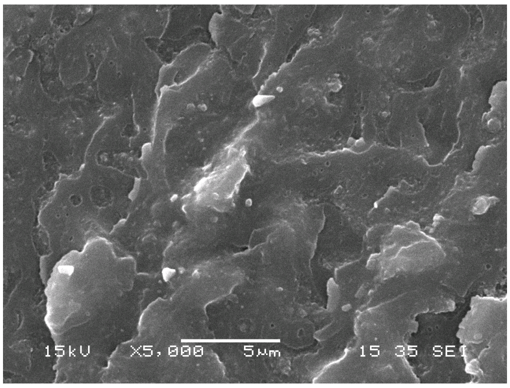
25.9	37.0	37.0	35–50–50<br>(III)	35.8	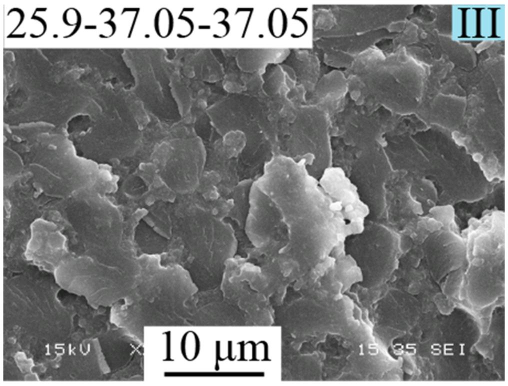

27.3	36.4	36.4	37.5–50–50 (III)	35.8	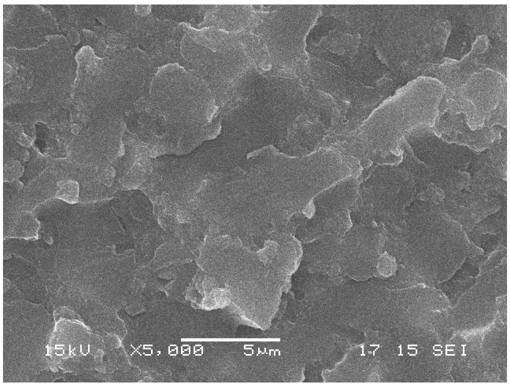
28.6	35.7	35.7	40–50–50 (I)	35.8	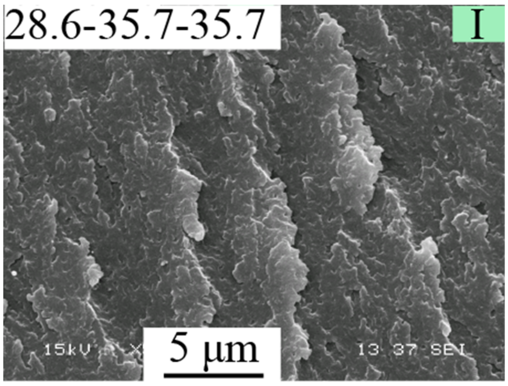
33.3	33.3	33.3	50–50–50 (I)	35.8	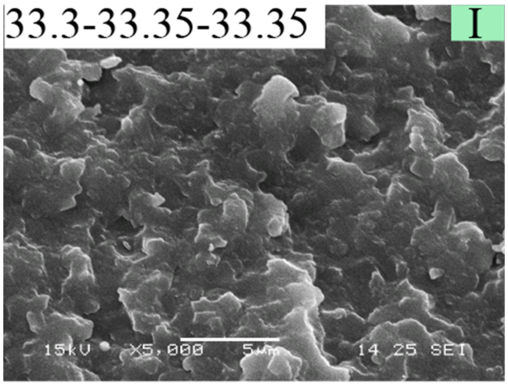
9.1	40.9	50.0	10–45–55 (II)	35.5	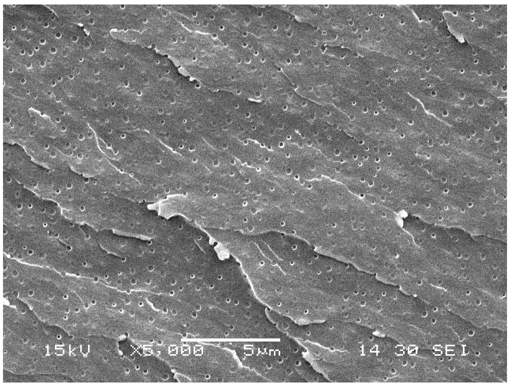

16.7	37.5	45.8	20–45–55<br>(II)	35.5	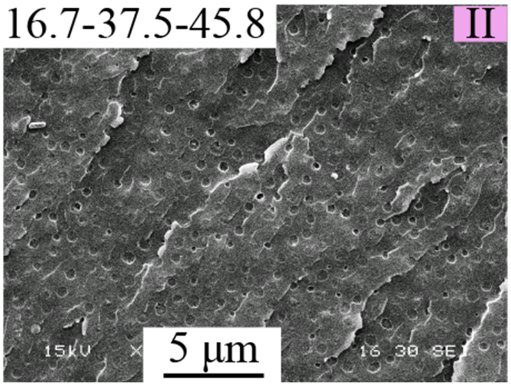
20.0	36.0	44.0	25–45–55<br>(III)	35.5	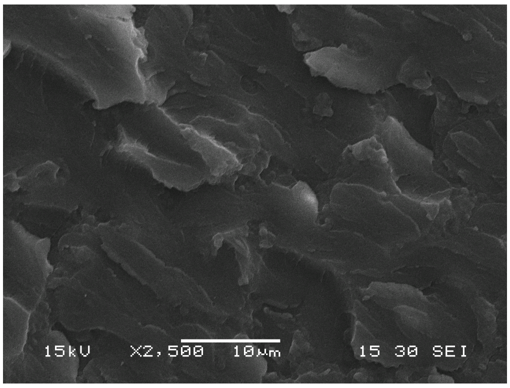
23.1	34.6	42.3	30–45–55<br>(III)	35.5	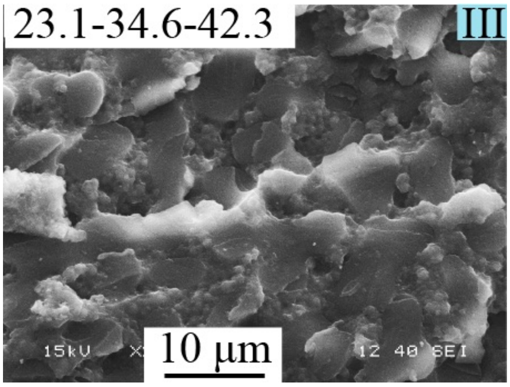
25.9	33.3	40.7	35–45–55<br>(IV)	35.5	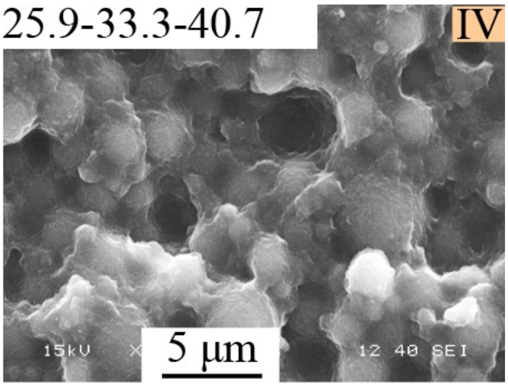

16.7	33.3	50.0	20–40–60 (II)	35.2	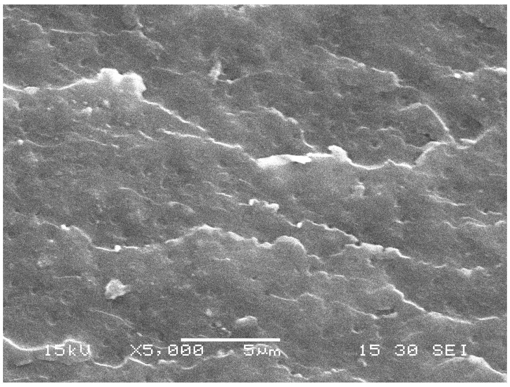
20.0	32.0	48.0	25–40–60 (III)	35.2	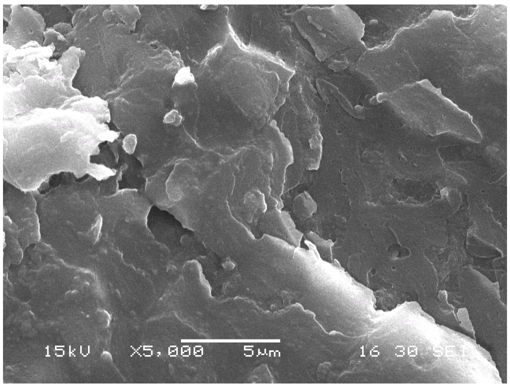
23.1	30.8	46.2	30–40–60 (III)	35.2	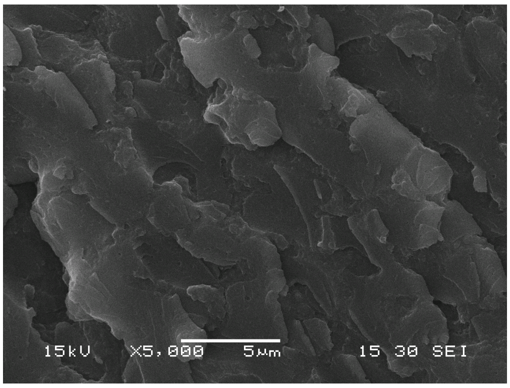
25.9	29.6	44.4	35–40–60 (IV)	35.2	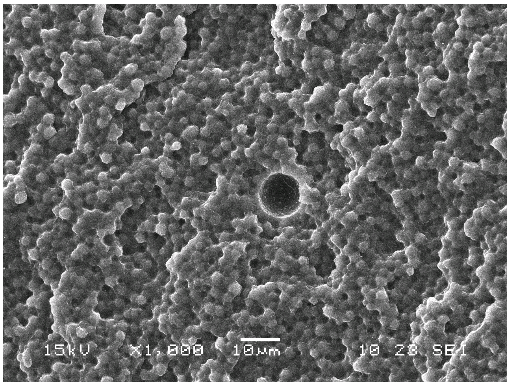

16.7	29.2	54.2	20–35–65 (I)	34.9	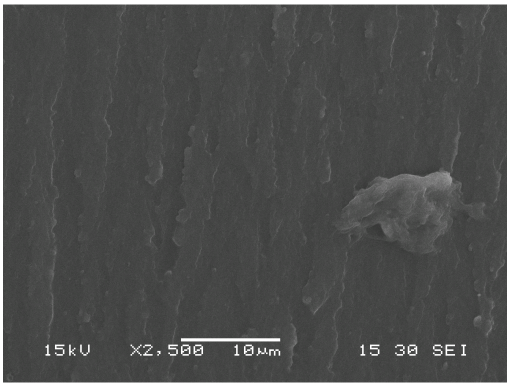
23.1	26.9	50.0	30–35–65 (I)	34.9	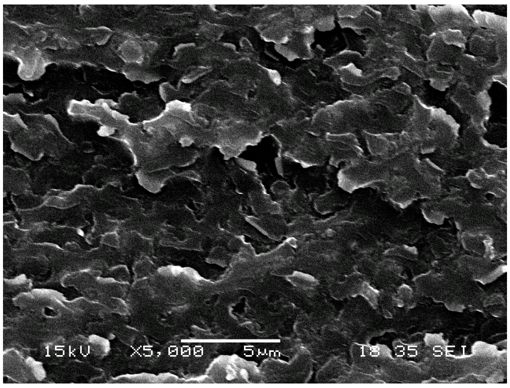
28.6	25.0	46.4	40–35–65 (I)	34.9	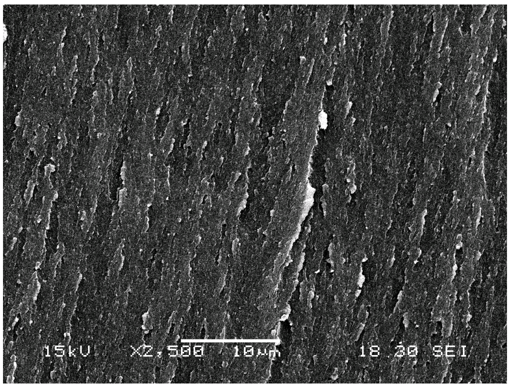
33.3	23.3	43.3	50–35–65 (I)	34.9	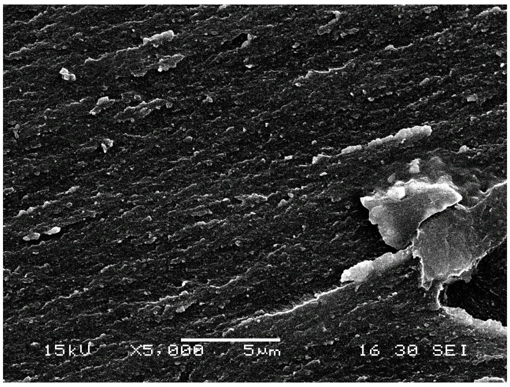
